# Identification of a novel amphioxus leucine-rich repeat receptor involved in phagocytosis reveals a role for Slit2-N-type LRR in bacterial elimination

**DOI:** 10.1016/j.jbc.2023.104689

**Published:** 2023-04-10

**Authors:** Yanli Zhan, Chen-si Zhao, Xuemei Qu, Zhihui Xiao, Chong Deng, Yingqiu Li

**Affiliations:** MOE Key Laboratory of Gene Function and Regulation, Guangdong Province Key Laboratory of Pharmaceutical Functional Genes, State Key Laboratory of Biocontrol, School of Life Sciences, Sun Yat-sen University, Guangzhou, China

**Keywords:** amphioxus, folate receptor domain (Fr domain)-containing leucine-rich repeat receptor (FrLRR), Grb2, Slit2-N, bacterial binding, phagocytosis

## Abstract

The basal chordate amphioxus is a model for tracing the origin and evolution of vertebrate immunity. To explore the evolution of immunoreceptor signaling pathways, we searched the associated receptors of the amphioxus *Branchiostoma belcheri* (Bb) homolog of immunoreceptor signaling adaptor protein Grb2. Mass-spectrum analysis of BbGrb2 immunoprecipitates from *B. belcheri* intestine lysates revealed a folate receptor (FR) domain- and leucine-rich repeat (LRR)-containing protein (FrLRR). Sequence and structural analysis showed that FrLRR is a membrane protein with a predicted curved solenoid structure. The N-terminal Fr domain contains very few folate-binding sites; the following LRR region is a Slit2-type LRR, and a GPI-anchored site was predicted at the C-terminus. RT-PCR analysis showed FrLRR is a transcription-mediated fusion gene of BbFR-like and BbSlit2-N-like genes. Genomic DNA structure analysis implied the *B. belcheri* FrLRR gene locus and the corresponding locus in *Branchiostoma floridae* might be generated by exon shuffling of a Slit2-N-like gene into an FR gene. RT-qPCR, immunostaining, and immunoblot results showed that FrLRR was primarily distributed in *B. belcheri* intestinal tissue. We further demonstrated that FrLRR localized to the cell membrane and lysosomes. Functionally, FrLRR mediated and promoted bacteria-binding and phagocytosis, and FrLRR antibody blocking or Grb2 knockdown inhibited FrLRR-mediated phagocytosis. Interestingly, we found that human Slit2-N (hSlit2-N) also mediated direct bacteria-binding and phagocytosis which was inhibited by Slit2-N antibody blocking or Grb2 knockdown. Together, these results indicate FrLRR and hSlit2-N may function as phagocytotic-receptors to promote phagocytosis through Grb2, implying the Slit2-N-type-LRR-containing proteins play a role in bacterial binding and elimination.

Amphioxus, a basal chordate occupying the transitional position between invertebrates and vertebrates, has been demonstrated to be an important model for studying the origin and evolution of the immune system (1, 2, 3). One of the efficient ways to explore the unknown amphioxus immune receptors is using the amphioxus homologs of the known vertebrate receptor-proximal key signaling proteins which are normally recruited to the receptor complex as bait to fish their binding receptors (2). The growth factor receptor-bound protein 2 (Grb2) is a receptor-proximal adaptor protein in both antigen receptor signaling and innate immune signaling and plays an important role primarily by nucleating signalosomes *via* its Src Homology(SH) 2 domain and SH3 domains to transduce signaling pathways and reassemble the cytoskeleton (4, 5). The interaction between Grb2 SH3 domains and Wiskott–Aldrich Syndrome protein (WASP) plays a role in regulating the actin cytoskeleton (6). The heterotrimer phospholipase D2-Grb2-WASp enables actin nucleation at the phagocytic cup and promotes phagocytosis, which is at the center of innate immune system function (7). The interaction between Grb2 and the cellular prion protein tail also initiates cytoskeletal rearrangement, inducing generalized membrane ruffling that drives bacterial swimming internalization, as microbes ride the wave of the membrane until they are enclosed within macropinosomes (8). Grb2 is also involved in Fc gamma receptor (FcγR) clustering-induced phagocytosis (9, 10). During *Mycobacterium tuberculosis* infection, human mannose receptor signaling temporally regulates phagocytosis and phagosome-lysosome fusion through Grb2 (11). In T cells, upon T cell receptor (TCR) activation, Grb2 is recruited to the TCR signaling complex by the adaptor protein, the linker for activation of T cells, and Grb2 further recruits son of sevenless and WASP to activate the RAS pathway and promote actin assembly, respectively (4, 12). In B cells, after membrane-bound immunoglobulin G (mIgG)-B cell receptor (BCR) recognizes antigens, Grb2 is recruited to the cytoplasmic tail of mIgG. Grb2 in turn brings along the Tec family kinases Btk into the BCR signalosome to stabilize the BTK/BLNK/PLCγ2 complex and link BCR microclusters and motor proteins, leading to the remodeling of F-actin (13, 14). A homolog of Grb2 in amphioxus *Branchiostoma belcheri* (Gene id: 022850R, http://ac.agrogene.ac.cn/lancelet/) is documented. Based on the important roles of Grb2 in both adaptive and innate immune receptor signaling, the identification of amphioxus *B. belcheri* Grb2-binding receptors may help us to understand the basal chordate immune system and the evolution of immune receptor signaling.

Leucine-rich repeat (LRR) proteins are a superfamily of proteins containing several LRR motifs that are well known to mediate protein–protein interactions (15). These proteins, with diverse functions, include immune receptors such as variable lymphocyte receptors (VLR) and Toll-like receptors (TLRs) and neuronal cell adhesion molecules such as Slit and Slit- and Trk-like protein (Slitrk) (16, 17). VLRs are unconventional adaptive immune receptors that generate diversity to recognize different antigens through gene conversion of LRRs in jawless vertebrates (18, 19). VLR’s LRR region usually consists of N-terminal LRR (LRRNT), multiple LRRV (variable leucine-rich repeat) motifs, a connecting peptide (CP), C-terminal LRR (LRRCT), and the stalk region. LRRNT and LRRCT localize at the two ends of the LRRVs to protect the hydrophobic protein core region, similar to other typical LRR proteins, such as TLRs (20, 21). The curved solenoid structure of all three VLR isotypes, similar to TLRs, has been confirmed (22). The crescent-shaped VLR solenoid has high specificity to proteins and glycans, which is provided by a highly variable concave binding surface and the LRRCT insert (23). LRR and fibronectin type-III domain-containing protein, a homolog of VLR, was found to recognize bacteria and promote hemocytic phagocytosis (24). The TLR family senses the molecular signatures of microbial pathogens and plays a fundamental role in innate immune responses. TLRs are type I membrane proteins characterized by an ectodomain composed of LRRs that are responsible for the recognition of pathogen-associated molecular patterns and a cytoplasmic domain homologous to the cytoplasmic region of the interleukin-1 (IL-1) receptor, known as the Toll/interleukin-1 receptor (TIR) homology domain, which is required for downstream signaling (25). Slit is a secreted protein that was initially found in *Drosophila* and has the well-known function of ensuring correct axon growth (26). Slit consists of multiple domains, including four LRR regions (LRR region: an LRRNT, several LRR motifs and an LRRCT), 9 EGF repeats, a laminin G–like domain, and a C-terminal cysteine knot (27). The slit can be cleaved into the N-terminus (Slit-N), which contains four LRR regions and five EGF repeats, and the C-terminus (Slit-C), which contains the remaining domains (28). Mammalian Slit has three isoforms identified thus far; together with their transmembrane Roundabout (Robo) receptors, they act as neuronal guidance cues that guide neuronal axon path branching and control neuronal migration by regulating actin cytoskeletal rearrangements (29). Slit2 has been shown to function as chemorepellent in immune cells to inhibit their directed migration toward chemotactic stimuli by regulating actin networks (30). Recently, it is reported that Slit2 inhibits macropinocytosis by inducing cytoskeletal changes in mice macrophages (30); and Slit2 has a role in regulating phenotypic plasticity of tumor-associated macrophages, in which Slit2 suppresses IL-6 production in the tumor microenvironment and then leads to activation of M1-like phagocytic and anti-fibrotic macrophages (31). To date, whether the LRR regions of Slit2 or other Slits could function like TLR/VLR to recognize pathogens is unknown.

The folate receptors (FRs) bind to folate and reduced folic acid derivatives and mediate the delivery of 5-methyltetrahydrofolate to the interior of cells (32). The FRs exhibit the spherical structure, in which five α-helices and four β-strands form the folate receptor domain (Fr domain) with the folate binding pocket consisting of 15 residues involved in folate binding (33, 34, 35). Human FRs consist of four isoforms 1 to 4, and except hFR3, are glycosyl-phosphatidylinositol (GPI)-anchored cell membrane proteins (35, 36). The molecular functions and biological effects of the four FR isoforms are different, which is closely related to the respective organizations they express (32). FR1 is highly expressed in some cancer cells proliferating rapidly and therefore becomes a target of diagnosis and treatment to a range of solid tumors (37). FR2 is a differentiation marker in normal hematopoiesis and is unable to bind folate in normal hematopoietic cells; in contrast, it binds folate in the placenta (32). FR3 is primarily a secretory protein due to the lack of an efficient signal for GPI modification, and FR3 may be useful as a serum marker of lymphoid tumors as it is virtually undetectable in normal serum (32). FR4 is highly expressed on the surface of murine regulatory T cells (Treg) and has an impact on the proliferation and function of Treg (38). However, it was later discovered that FR4 may not bind folate because several key amino acids that mediate folate binding are not conserved in FR4 (39), and among them, the four amino acids of hFR1 or hFR2 that form the key hydrogen bond with folate (aspartic acid, tryptophan, arginine, and histidine) are replaced by alanine, glycine, glutamine, and arginine in hFR4 (40).

In this study, using amphioxus *B. belcheri* Grb2 as a bait, we revealed a Slit2-type LRR region-containing and BbGrb2-associated receptor, FrLRR, which is mostly distributed in the *B. belcheri* intestine. The predicted tertiary structure of FrLRR is a typical curved solenoid structure like VLRs. We proved that the FrLRR is a readthrough transcript of a BbFR-like gene and a BbSlit-N-like gene and the transcripts of BbFR-like and BbSlit-N-like genes are also expressed. We predicted that the FrLRR gene locus in *B. belcheri* and the corresponding locus in *Branchiostoma floridae* might be produced by exon shuffling of Slit2-N LRR region into a FR-like gene. We further demonstrated that FrLRR promotes bacterial binding to the cell surface and phagocytosis through Grb2. This function disappeared if either the Fr domain or the LRR region in FrLRR was deleted, indicating that the readthrough transcript of BbFR-like and BbSlit-N-like genes produces a novel functional receptor. Moreover, we revealed that overexpression of human Slit2-N which contains the homolog LRR region with FrLRR also directly promotes bacterial binding to the cell surface and phagocytosis *via* Grb2. Thus, this study discovered a novel amphioxus *B. belcheri* immune receptor, FrLRR, and revealed a previously undescribed potential role for Slit2-N-type LRR-containing proteins in bacterial binding and elimination.

## Results

### FrLRR coimmunoprecipitates with BbGrb2

**Figure 1:**
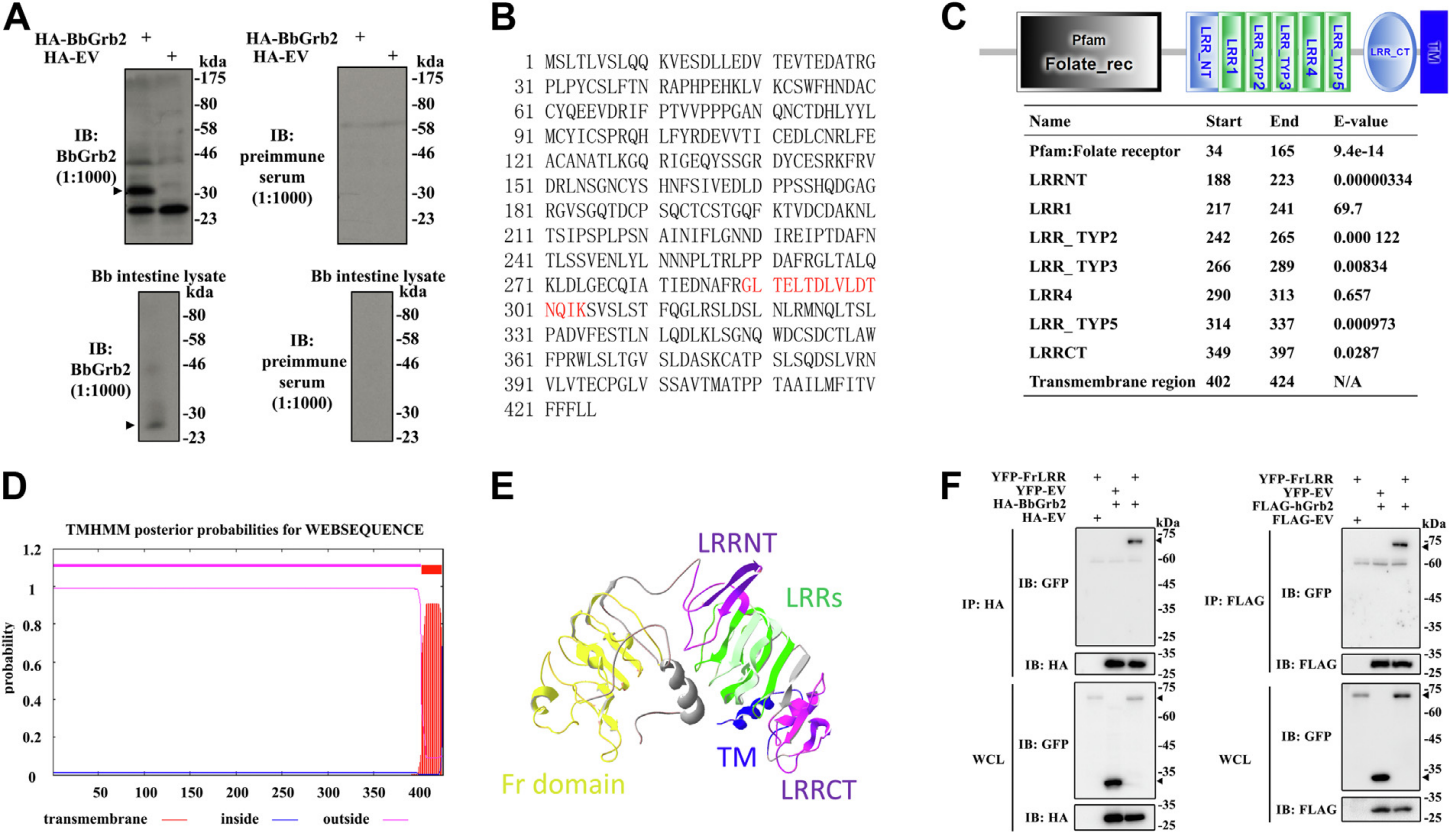
**FrLRR coimmunoprecipitates with BbGrb2.***A*, the efficiency of the anti-BbGrb2 polyclonal antibody (titer 1:1000) was determined in HEK293T cells overexpressing HA-BbGrb2 (*upper panels*) and in amphioxus *B. belcheri* intestine lysate (*lower panels*). *Black arrowheads* indicate the position of target protein HA-BbGrb2 or endogenous Bb-Grb2. *B*, the amino acid sequence of FrLRR. Peptide identified by mass spectrometry was highlighted in red. *C*, protein domains of FrLRR. *D*, transmembrane region of FrLRR predicted by TMHMM. The predicted transmembrane region (23 amino acid residues) is located in the C-terminus of FrLRR. *E*, predicted 3D structure of FrLRR. *F*, FrLRR interacts with BbGrb2 or hGrb2 when overexpressed. HEK293T cells transfected with YFP-FrLRR together with HA-EV (empty vector), HA-BbGrb2 or FLAG-EV, or FLAG-hGrb2 were lysed and immunoprecipitated with anti-HA or anti-FLAG antibody and then immunoblotted with anti-GFP, anti-HA and anti-FLAG antibodies. Data are representatives of at least two independent experiments. 3D, three-dimensional; EV, empty vector; Folate_rec, folate receptor domain, Fr; Fr domain, folate receptor domain; FrLRR, Fr domain-containing leucine-rich repeat receptor; IB, immunoblot analysis; IP, immunoprecipitation; LRR, leucine-rich repeat; LRRCT, C-terminal LRR; LRRNT, N-terminal LRR; LRR_TYP, leucine-rich repeats, typical (most populated) subfamily; TM, transmembrane region; WCL, whole cell lysates.

To determine whether any immune receptor was associated with Grb2 in amphioxus *B. belcheri*, we first cloned the DNA fragment of the BbGrb2 gene from the *B. belcheri* intestinal cell complementary deoxyribonucleic acid (cDNA). BbGrb2 has 71.41% nucleotide and 74.77% amino acid identity, respectively, with *Homo sapiens* Grb2 (hGrb2). Next, we prepared a murine polyclonal antibody against BbGrb2 and verified its recognition specificity for BbGrb2 (Fig. 1*A*). Using this homemade anti-BbGrb2 antibody, we immunoprecipitated the BbGrb2 protein complex from the lysates of amphioxus *B. belcheri* intestinal cells stimulated by phorbol myristate acetate (PMA) and ionomycin to mimic immune receptor stimulation (41) and analyzed the anti-BbGrb2 immunoprecipitates by mass spectrometry. The ‘GLTELTDLVLDTNQIK’ peptide was detected by mass spectrometry, and the complete amino acid and nucleic acid sequences of this protein were obtained by alignment in the amphioxus genome database (http://ac.agrogene.ac.cn/lancelet/, transcription ID. 022630R.t2) (Figs. 1*B* and S1). We then cloned this target gene by reverse transcription PCR (RT-PCR) from *B. belcheri* intestinal total RNA. Analysis using the SMART online tool showed that this protein contains a Fr domain and multiple LRR motifs (Fig. 1*C*), and so we named it FrLRR.

Analysis using the SMART and TMHMM online tools revealed that from the N-terminus to the C-terminus in FrLRR, the predicted domains were a Fr domain, an LRRNT, an LRR1, four LRRVs, an LRRCT, and a transmembrane domain (Fig. 1, *C* and *D*). Moreover, the predicted tertiary structure of FrLRR is a curved solenoid structure, which resembles the typical characteristics of VLRs. The sequences of LRRVs are conserved and follow the rule of LRR motif (XLXXLXXLXLXXNXLXXLPXXXFX, X represents any amino acid) (42). Each LRRV of FrLRR is a β-sheet, and four β-sheets connect to form the concave surface of the solenoid structure (Fig. 1*E*), which might mediate the recognition and binding of ligands like LRR region-containing receptors, such as VLRs (42, 43, 44). Only a single peptide derived from FrLRR (the protein coverage is 2.57%) was detected by mass spectrometry analysis of Grb2 immunoprecipitates while 497 other peptides were revealed in total (Table S2). We further validated the association between FrLRR and BbGrb2 by cotransfection of FrLRR with BbGrb2 or hGrb2 in HEK293T cells. Coimmunoprecipitation showed that FrLRR is associated with BbGrb2 or hGrb2 (Fig. 1*F*). This result is consistent with the conclusion that when analyzing mass spectrometry data, protein identifications based on single peptides should be treated *at par* with identifications based on multiple peptides because many of the single-hit proteins are actually positive (45). Thus, FrLRR is a receptor that could recruit BbGrb2 and might function in the immune response.

### Homology analysis of FrLRR

**Figure 2:**
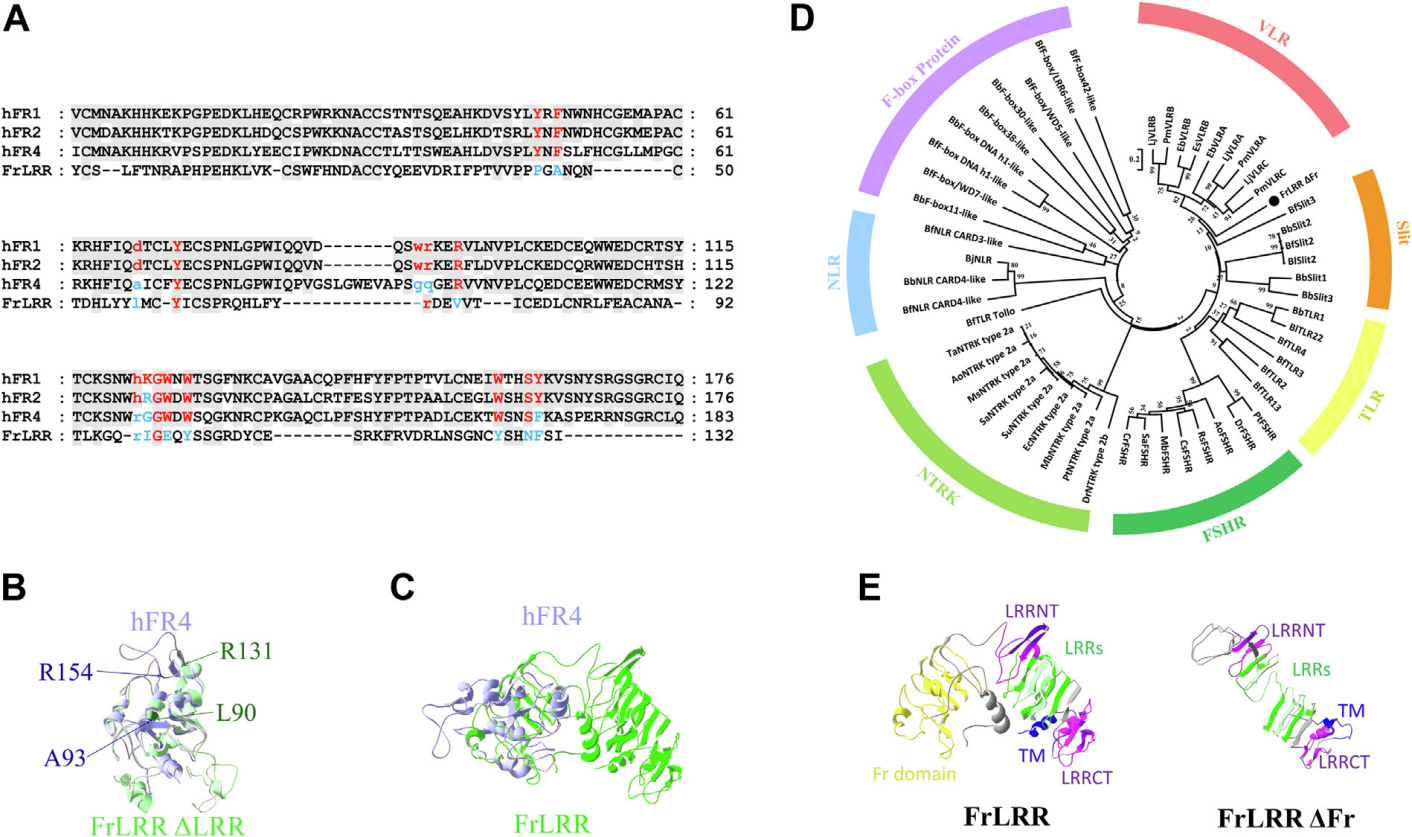
**Homology analysis of FrLRR.***A*, the Fr domain alignment of hFR1, hFR2, hFR4, and FrLRR. Letters on a *gray background* indicate identical amino acids; Letters in *red* indicate residues locate in the FA binding pocket and mediate FA binding; *Blue letters* in hFR4/FrLRR indicate the different amino acids in the corresponding position to FA-binding pocket of hFR1/2. *Red letters* in the *lower case* indicate residues to form hydrogen bonds to anchor folate in hFR1 and 2, and the corresponding residues in hFR4 and FrLRR are also indicated by lower case letters. *B*, superposition of predicted 3D structures of FrLRR ΔLRR (*green*) and hFR4 (*slate blue*) by the Phyre2 software. The residues labeled in *dark slate blue* mean two of the four amino acids in hFR1 or hFR2 that form the key hydrogen bond with folate, and those in *dark green* indicate the counterpart in FrLRR. *C*, superposition of predicted 3D structures of full-length FrLRR (*green*) and hFR4 (*slate blue*) by the Phyre2 software. *D*, molecular phylogenetic analysis of FrLRR in various species. The phylogenetic tree was constructed using MEGA 5.0 according to calculations with the neighbor-joining method (500 bootstraps). The numbers at the nodes indicate bootstrap values. *E*, comparison of the predicted 3D structures between full-length FrLRR and FrLRR ΔFr. FA, folic acid; F-box protein, proteins containing F-box domain and other domains such as LRR and WD repeat domain; FrLRR, Fr domain-containing leucine-rich repeat receptor; FSHR, follicle-stimulating hormone receptor, containing LRRs, a hinge region, and the transmembrane domain; NLR, nucleotide-binding domain and LRR-containing receptor, consist of an N-terminal CC or TIR domain, a central nucleotide-binding domain and a C-terminal LRR domain; NTRK, neurotrophic tyrosine kinase receptor, comprised of three LRR domains, two Ig domains and a tyrosine kinase domain.

Since FrLRR is predicted to contain a folate receptor domain at the N-terminus and a predicted transmembrane region, we first aligned the amino acid sequence and the 3D structure of FrLRR's Fr domain with those of the three membrane-anchored FR isoforms hFR1, hFR2, and hFR4. The results showed that the Fr domain of FrLRR has 26.02%, 25.2%, and 25.55% amino acid identity with hFR1, 2 and 4, respectively (Fig. 2*A*). However, like in FR4, the key amino acid residues mediating folate binding in the Fr domain (amino acids in red, Fig. 2*A*) were also less conserved in FrLRR (35, 36) (Fig. 2*A*), and the two amino acids aspartic acid and histidine of the four amino acids (D, W, R, H, highlighted by red letters in lower case in Fig. 2*A*) that form the key hydrogen bond with folate in hFR1 and 2 have been replaced by leucine and arginine in FrLRR (L90 and R131 in full-length sequence) (Fig. 2*A*). Further, the predicted three-dimensional (3D) protein structure alignment of FrLRR ΔLRR (deleting the LRR region that starts from LRRNT to LRRCT) (Fig. S2) with hFRs revealed the superimposition of the Fr domains (Figs. 2*B* and S3). However, a large change occurred in the predicted spatial position of R131 in FrLRR ΔLRR, compared with the corresponding R154/hFR4, R125/hFR1, or R119/hFR2 (Figs. 2*B* and S3). The R131 of FrLRR ΔLRR locates in a small loop between two α-helices, while the corresponding arginines of hFRs locate in a big loop between two β-sheets (Figs. 2*B* and S3). The predicted spherical structure of the Fr domain in FrLRR ΔLRR disappeared when in full-length FrLRR because the secondary structure changed, which led to a completely different tertiary structure of FrLRR's Fr domain from hFRs (Figs. 2*C*, S3 and S4). Along with these, the spatial positions of the key folate-binding amino acid residues distributed in these secondary structures also changed markedly (Fig. S4). These results suggest that although the Fr domain alone of FrLRR has an amino acid identity and structure similarity with hFR1, 2 and 4, it could not bind folate due to the changes in the key folate binding sites and the very different predicted tertiary structure in FrLRR from hFRs.

Since FrLRR has an LRR region in its C terminus and to understand which type of LRR protein FrLRR is, we constructed a phylogenetic tree by using various LRR proteins from different species (Fig. 2*D*). The result showed that the LRR region-containing fragment of FrLRR was clustered into the VLR group and adjacent to the Slit group. Thus, FrLRR might be evolutionarily close to the VLRs. If the Fr domain is deleted, the tertiary protein structure of FrLRR's LRR region (FrLRR ΔFr) decreases the bending degree, and the spatial arrangement of four β-sheets become loose, which may disrupt the favorable structure for recognizing ligands (Fig. 2*E*). This suggests that the presence of the Fr domain might help the LRR region of FrLRR form a protein structure that is more conducive to ligand binding.

### FrLRR is a unique gene that might only be present in amphioxus *B. belcheri*

**Figure 3:**
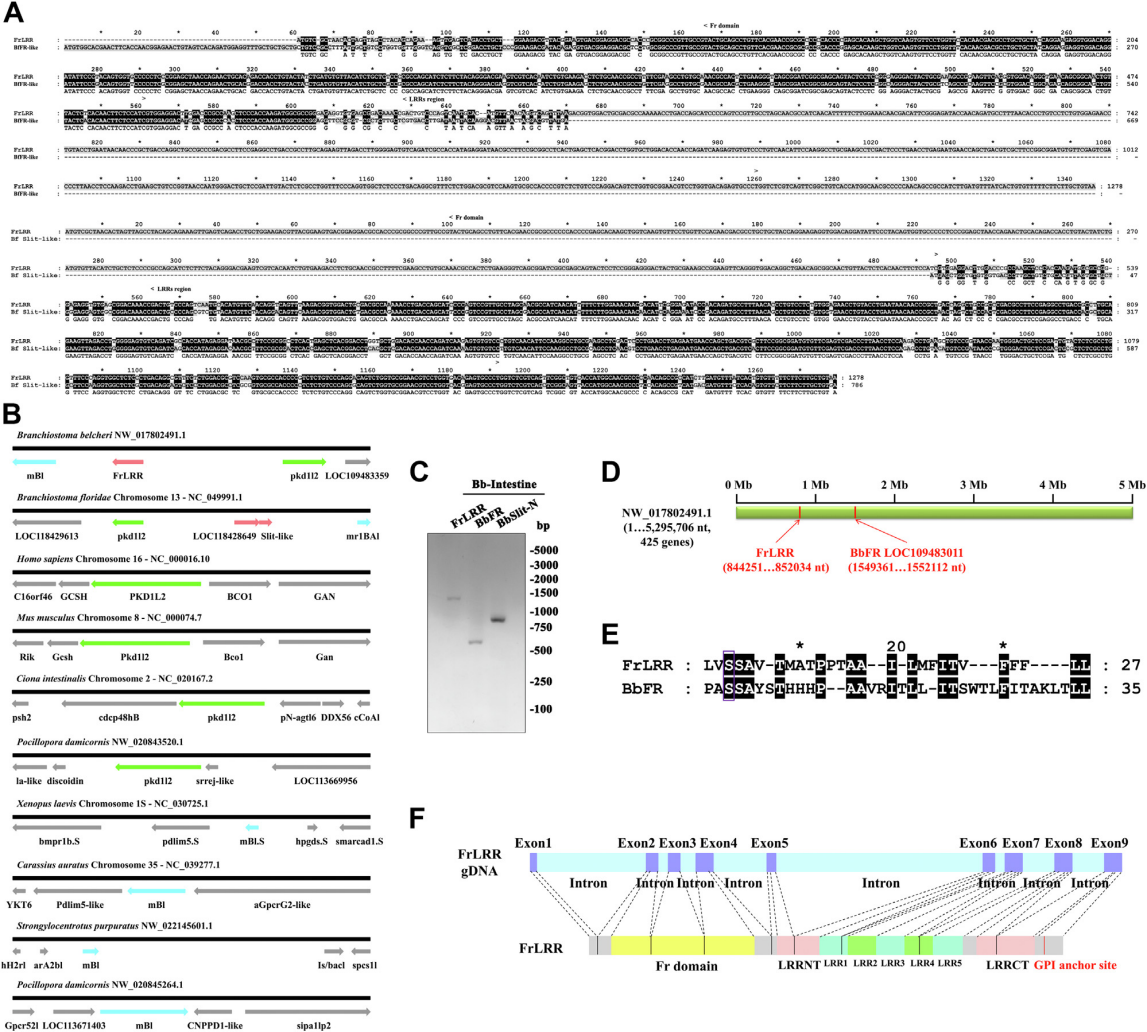
**FrLRR is a unique gene that is only present in amphioxus.***A*, gene alignment of FrLRR with LOC118428649 (BfFR-like) or LOC118429728 (BfSlit-N-like). GenBank accession numbers: LOC118428649 (XM_035838758), LOC118429728 (XM_035840346). *B*, schematic diagram of syntenic regions of FrLRR in other species. The *black horizontal line* represents the genomic chromosomes or scaffolds. The *pink arrows* indicate the genome sequences of FrLRR and BfFR-like/Slit-N-like, and the *green* and *blue arrows* indicate the genome sequences of pkd1l2 and mBl, respectively. The nonsyntenic genes among species are shown as *gray arrows*. *C*, RT-PCR analysis confirms that all three transcripts BbFR-like, BbSlit2-N-like, and FrLRR are expressed in *B. belcheri*. FrLRR is 1278 bp, BbFR-like (LOC109483991) is 666 bp, and BbSlit2-N-like (LOC109483727) is 786 bp. Total RNA was isolated from the intestine of one *B*. *belcheri*. The primer sequences can be found in Table S1. *D*, schematic diagram of the location of FrLRR and BbFR LOC109483011 on amphioxus *B. belcheri* genomic scaffold 11. The *green horizontal block* represents genomic scaffold 11 (NW_017802491.1), in which two *red vertical bars* indicate the genomic positions of FrLRR at nucleotides 844251-852034 and the BbFR LOC109483011 at nucleotides 1549361-1552112, respectively. *E*, comparison of the GPI-anchor site and C-terminal hydrophobic region in FrLRR with its counterparts in BbFR. The residues in the frame indicate the predicted GPI-anchor sites with the best score. Letters on a *black background* indicate identical residues. *F*, schematic diagram of exons encoding FrLRR. The *slate blue bars* mean exons; the *light cyan bars* mean introns; the *light grey* bars mean sequences with no predictable functional domains; the *yellow* bar means the Fr domain; the *pink bars* mean LRRNT and LRRCT; the *light green* and *cyan bars* mean LRRs. The GPI anchor site is marked in *red*. FrLRR, Fr domain-containing leucine-rich repeat receptor.

To explore the evolution of the FrLRR gene and considering that the *B. belcheri* FrLRR gene should also exist in *B. floridae*, we searched for homologs of the FrLRR gene in the amphioxus database (http://ac.agrogene.ac.cn/lancelet/). Surprisingly, on *B. floridae* chromosome 13, the homologous genome fragment has been predicted to encode two independent but closely adjacent genes, the Bf_85164 (or LOC118428649 in NCBI) gene (encoding an FR-like protein without a GPI anchor site at the C-terminus; we named it BfFR-like) and a Slit-like gene (Bf_85165 or LOC118429728 in NCBI, encoding only an LRR region of Slit-N-like protein, as shown in Fig. S5*A*; we named it BfSlit-N-like), exhibiting high (90% and 87%) nucleotide identity with the Fr domain and LRR region of FrLRR, respectively (Fig. 3*A*). Furthermore, we compared FrLRR and its syntenic genes in flanking regions to the genomes of various species. The upstream syntenic gene of FrLRR on scaffold 11 is the polycystic kidney disease 1 like 2 gene (PKD1L2), and downstream is the melanopsin-B-like protein (mBl). We selected the genomic regions containing these two genes from different species of vertebrates and invertebrates and compared them to those of FrLRR and found that only *B. floridae* has a similar genome structure, that is, the pkd1l2 gene localizes upstream of the LOC118428649 gene (BfFR-like) and the mBl gene downstream of the BfSlit-N-like gene (Fig. 3*B*). These results indicate that the structure of the genome fragment harboring FrLRR or BfFR-like/Slit-N-like only exists in amphioxus. Because both the FR and Slit genes are very ancient genes that already existed from protozoa and tardigrada, respectively, it is possible that FrLRR is a fusion gene between a Fr domain-like gene and a Slit-N-like gene.

Since we have cloned FrLRR from *B. belcheri* cDNA, and the amphioxus database website (http://ac.agrogene.ac.cn/lancelet/) annotates FrLRR as a single gene (Gene ID: 022630R and transcript ID: 022630R.t2) in *B. belcheri*; thus, we have proposed that FrLRR is a single gene. However, in the NCBI gene database, both the corresponding positions of FrLRR in *B. belcheri* and in *B. floridae* are annotated as two separate genes (a FR-like gene and a Slit2-N-like gene; LOC109483991 and LOC109483727 for *B*. *belcheri*, LOC118428649 and LOC118429728 for *B. floridae*). Thus, the FrLRR transcript may be produced by a readthrough transcription of the two adjacent genes, which has been reported (46). By using the specific primers for *B. belcheri* FrLRR, LOC109483991, and LOC109483727 to performing RT-PCR, we obtained all three transcripts and confirmed them as FrLRR, BbFR-like, and BbSlit2-N-like by sequencing (Figs. 3*C*, S6 and S7). This result indicated that FrLRR is produced by a readthrough transcript of two separate genes, a BbFR-like and a BbSlit2-N-like gene. The finding of FrLRR sets a new example for the creation of new gene structures through the transcription-mediated gene fusion mechanism (46).

To find out how the readthrough transcript of FrLRR was generated, we analyzed the genome sequence of the FrLRR gene locus and compared it with the corresponding genome sequence containing BfFR-like and BfSlit2-N like genes in *B. floridae* (Fig. S8), we noticed a pair of canonical splice donor and acceptor motifs (GT, AG) exists in the intron of FrLRR fused gene between exon 4 and exon 5 (which is also the intergenic region containing the stop codon of the upstream BbFR like gene) and thus can splice out the translation stop codon of the upstream gene to generate a functional fused transcript (Fig. S8). Of note, in *B*. *floridae*, the correspondence splicing acceptor motif is mutated to GG, and an FrLRR-similar readthrough transcript has not been recorded in the *B. floridae* transcriptomics database (http://ac.agrogene.ac.cn/lancelet/); therefore, whether it is generated is unknown. It is reported that stress can induce readthrough transcription (47), and further understanding of the mechanism for the FrLRR transcript generation will help to delineate the evolution of the amphioxus immune system.

To understand the evolutionary generation of FrLRR gene locus and the corresponding position in *B. floridae*, we searched the *B. belcheri* database (Transcripts/B.belcheri_v18h27.r3_ref_cds) and found that the gene with the highest sequence identity to the Fr domain of FrLRR (75%) was LOC109483011. This gene (669 bp) encodes an FR-like protein containing a GPI anchor site at the C-terminus, which we named BbFR here. To determine whether the Fr domain of FrLRR was copied from BbFR, we first determined whether they are located on the same genome scaffold. By searching the *B. belcheri* genome database (Genomes/B.belcheri_v18h27.r3_ref_genome), we found that FrLRR is located on nucleotides 844251-852034 of scaffold 11 (the sequence number on NCBI is NW_017802491.1), while BbFR is also located on scaffold 11, nucleotides 1549361-1552112 (Fig. 3*D*). Interestingly, in *B. floridae*, the gene LOC118429252 (encoding an FR-like protein containing a GPI anchor site at the C-terminus; we named it BfFR), which has 56% sequence identity with *B. floridae* LOC118428649 (BfFR-like) in protein sequences, is on the same chromosome (number 13) as the BfFR-like gene. Thus, the Fr domain of FrLRR might be a copy of BbFR. Interestingly, both BbFR and FrLRR have a predicted and similar GPI anchor site at their C-terminuses which showed very low homologous with hFRs (Figs. 3*E* and S9). Thus, FrLRR may be a GPI anchor receptor, like FRs.

Unlike the Fr domain of FrLRR, which might be a copy of BbFR (LOC109483011) on scaffold 11, there is a Slit-N-like gene (LOC109484659, encoding two LRR regions, as shown in Fig. S5*A*) in scaffold 7 but not in scaffold 11 of *B. belcheri*. In *B. floridae*, in addition to the copy of Slit-N-like gene (LOC118429728) adjacent to BfFR-like in chromosome 13, the other copy of Slit-N-like gene (LOC118427592) is in chromosome 1. Amphioxus also has three isoforms of Slit-like genes, and BbSlit2 is on scaffold 44. The above three Slit-N-like genes have a high sequence identity with the amphioxus Slit2 gene (Fig. S5*B*). Further, we analyzed the exon layout of FrLRR and found that the FrLRR gene contains 9 exons (Fig. 3*F*): the Fr domain is encoded by exons 2 to 4, the following LRR region is encoded by exons 5 to 8, and exon 9 encodes the C-terminal 103 nucleotides of LRRCT and the C-terminal region harboring the GPI anchor site. Since the C-terminus following the LRR region of Slit does not contain a GPI anchor site, and amphioxus Slit2 and Slit2-N-like genes are not on the same genome scaffold with FrLRR, the FrLRR gene locus might be generated by the shuffling of several exons (encoding LRR regions) from Slit2 or Slit2-N-like genes into an FR gene locus. There are three major molecular mechanisms for exon shuffling: (1) transposon-mediated exon shuffling which requires transposable elements; (2) crossover during sexual recombination of parental genomes, in which exons from different genes insert into the introns of new genes positions; and (3) illegitimate recombination, in which a short homologous DNA sequence could mediate exon shuffling (46, 48). FrLRR gene locus does not contain the element for transposon mediating exon shuffling; thus, the first mechanism is not applicable to FrLRR. We further analyzed the gene structures of BbFR (LOC109483991), BfFR (LOC118428649), and hFRs. We noticed that they have a similar structure pattern in which the last single exon encodes the GPI anchor site and partial Fr domain (Fig. S10), implying if FrLRR gene locus is generated by the shuffling of Slit exons into BbFR gene locus, the exons should insert into the last exon of BbFR. Therefore, the second mechanism is neither applicable to FrLRR, but the mechanism of illegitimate recombination may explain our hypothesis that FrLRR might be generated by exon shuffling (Fig. S11). The finding of FrLRR gene locus and the corresponding position in *B. floridae* provides a new example for the report that amphioxus exhibits an active exon shuffling process that has made an essential contribution to their novel domain combination repertoire (49).

### FrLRR is distributed primarily in the amphioxus *B. belcheri* intestine and displays cytoplasmic membrane and lysosome localizations

**Figure 4:**
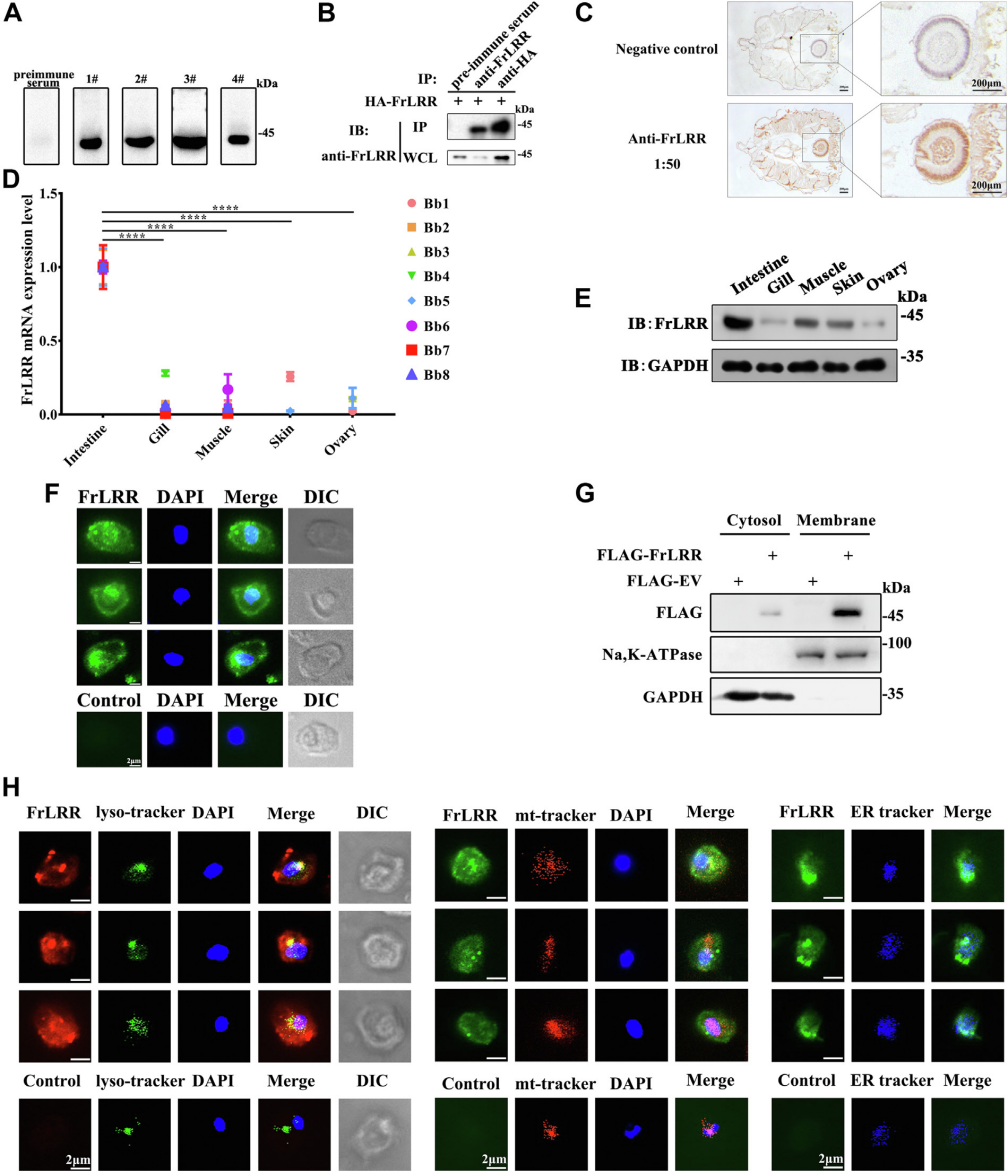
**FrLRR is primarily distributed in the amphioxus *B. belcheri* intestine and localizes to the cytoplasmic membrane and lysosomes.***A*, the efficiency of FrLRR polyclonal antibody was determined. Endogenous FrLRR was extracted from the amphioxus *B. belcheri* intestine and immunoblotted with preimmune serum and anti-FrLRR antibody. The preimmune serum and anti-FrLRR antibody were diluted 1000-fold. We prepared an anti-FrLRR antibody using four BALB/c mice labeled 1#-4#. *B*, immunoprecipitation of overexpressed FrLRR from the cell lysates of HEK293T cells using the homemade anti-FrLRR antibody from mouse 3#. The anti-FrLRR antibody was added to the cell lysates of HEK293T cells for immunoprecipitation, with preimmune serum and anti-HA antibody used as controls. The protein band of FrLRR was separated by SDS–PAGE and immunoblotted using the anti-FrLRR antibody. *C*, immunohistochemical staining analysis of the tissue distribution of FrLRR in amphioxus *B. belcheri*. Preimmune serum was used as a negative control. Scale bar, 200 μm. The cross sections of amphioxus *B. belcheri* after paraffin embedding are shown on the *left*, and *small black frames* indicate the intestine, which are magnified on the *right*. Scale bars, 200 μm. *D*, RT-qPCR analysis of the tissue distribution of FrLRR in the amphioxus *B. belcheri*. mRNA expression levels of endogenous FrLRR were calculated using the 2^−ΔΔCt^ method. ∗∗∗∗*p* < 0.0001 (two tailed, unpaired Student’s *t* test). The data are presented as the mean (± s.d.). The primer sequences can be found in Table S1. *E*, different tissue distributions of FrLRR. Homemade mouse anti-FrLRR antibody was used for immunoblotting. *F*, localization of endogenous FrLRR in intestinal cells of amphioxus *B. belcheri*. Amphioxus *B. belcheri* intestinal cells were extracted, bound to poly-L-lysine-coated coverslips, and then fixed. The cells were stained with anti-FrLRR polyclonal antibody followed by a secondary fluorescent antibody (Alexa Fluor 488 Goat Anti-Mouse IgG). Nuclei were stained with DAPI (4′,6-diamidino-2-phenylindole), a blue-fluorescent DNA stain. The cells and fluorescence were observed and imaged using fluorescence microscopy. Scale bars, 2 μm. *G*, cell fraction analysis of the subcellular localization of overexpressed FrLRR in HEK293T cells. FLAG and FLAG-FrLRR were overexpressed in HEK293T cells, which then underwent subcellular fractionation, and the subcellular localization of FrLRR was determined. Na^+^/K^+^-ATPase is a membrane-specific marker. GAPDH was used as an internal reference for protein expression in immunoblotting. *H*, immunostaining analysis of FrLRR localization in the lysosomes of amphioxus *B. belcheri* intestine cells. The intestinal cells of *B. belcheri* were labeled using three organelle dyes (LysoTracker-L7526, MitoTracker- M22426, ER-Tracker- E12353) according to the manufacturer’s guidelines. After fixation and permeabilization, the cells were incubated with anti-FrLRR antibody, using preimmune serum as a control. The primary antibody was then stained with the secondary antibody as in *F*. The cells and fluorescence were observed and imaged under a fluorescence microscope. Data are representatives of at least two independent experiments. DIC, differential interference contrast; FrLRR, Fr domain-containing leucine-rich repeat receptor; IB, immunoblot analysis; IP, immunoprecipitation; WCL, whole cell lysates.

To study the function of FrLRR, we prepared a mouse polyclonal antibody against FrLRR by immunizing four BALB/c mice with a fragment starting from the Fr domain to the LRRNT of FrLRR (36–223 aa). An obvious protein band near 45 kDa (the theoretical molecular mass of FrLRR is 46 kDa) in the amphioxus *B. belcheri* intestinal lysate was detected by all of these homemade anti-FrLRR antibodies but not by the preimmune serum, indicating that these anti-FrLRR antibodies are antigen-specific (Fig. 4*A*). Immunoprecipitation (IP) analysis showed that the homemade FrLRR antibody precipitated overexpressed HA-FrLRR from HEK293T cells (Fig. 4*B*).

To observe the tissue distribution of FrLRR in amphioxus *B. belcheri*, we performed immunohistochemical staining with the anti-FrLRR antibody. We found that FrLRR was primarily distributed in intestinal tissue (Fig. 4*C*). We further performed real-time PCR and western blotting to analyze the expression of FrLRR in different amphioxus *B. belcheri* tissues (Fig. 4, *D* and *E*) and found that BbFrLRR mRNA and protein were highly expressed in intestinal cells, as expected, but exhibited relatively low expression in gill, skin, muscle, and ovary cells. We further blotted the intestine lysates from different individuals and found that BbFrLRR had similar expression levels in them (Fig. S12*A*). These results demonstrate that FrLRR is a protein that is majorly expressed in the intestinal tissue of *B. belcheri*.

To confirm that FrLRR is a membrane receptor, we isolated amphioxus *B. belcheri* intestinal cells and conducted immunofluorescence staining using an anti-FrLRR antibody. We found that endogenous FrLRR exhibited both plasma membrane and cytoplasmic localization (Fig. 4*F*). Exogenously expressed FrLRR in HEK293T cells exhibited similar localization patterns (Fig. S12*B*). Consistently, subcellular fractionation analysis showed that overexpressed FrLRR primarily localized in the membrane of HEK293T cells with some cytoplasmic distribution (Fig. 4*G*). Of note, the FrLRR does not have a signal peptide. How does it go to the cell surface? It is possible that FrLRR belongs to a type of signal-anchor membrane protein, which does not have cleavable signal peptides but passes the membrane by their signal-anchor sequence, namely, a transmembrane helix (50). Considering that FrLRR also displayed a cytoplasmic distribution, we assessed the organelle localization of FrLRR. In primary amphioxus *B. belcheri* intestinal cells, the immunostaining results showed that endogenous FrLRR more obviously localized in lysosomes than in mitochondria or the endoplasmic reticulum (Fig. 4*H*). In HEK293T and Jurkat E6.1 cells, overexpressed FrLRR also displayed similar localization patterns (Fig. S13). Although we could not exclude the influence on the detection of FrLRR lysosomal localization by antibody cross-reacting with microbial peptides in lysosome or the overexpressed proteins-caused lysosomal location, the control antibodies from preimmune mice serum could not detect any FrLRR in intestine cells (Fig. 4*H*) may indicate FrLRR have real lysosome localization. If the staining is not artifactual, then the results suggest that FrLRR localizes to the plasma membrane and lysosomes.

### FrLRR enhances bacterial binding and phagocytosis

**Figure 5:**
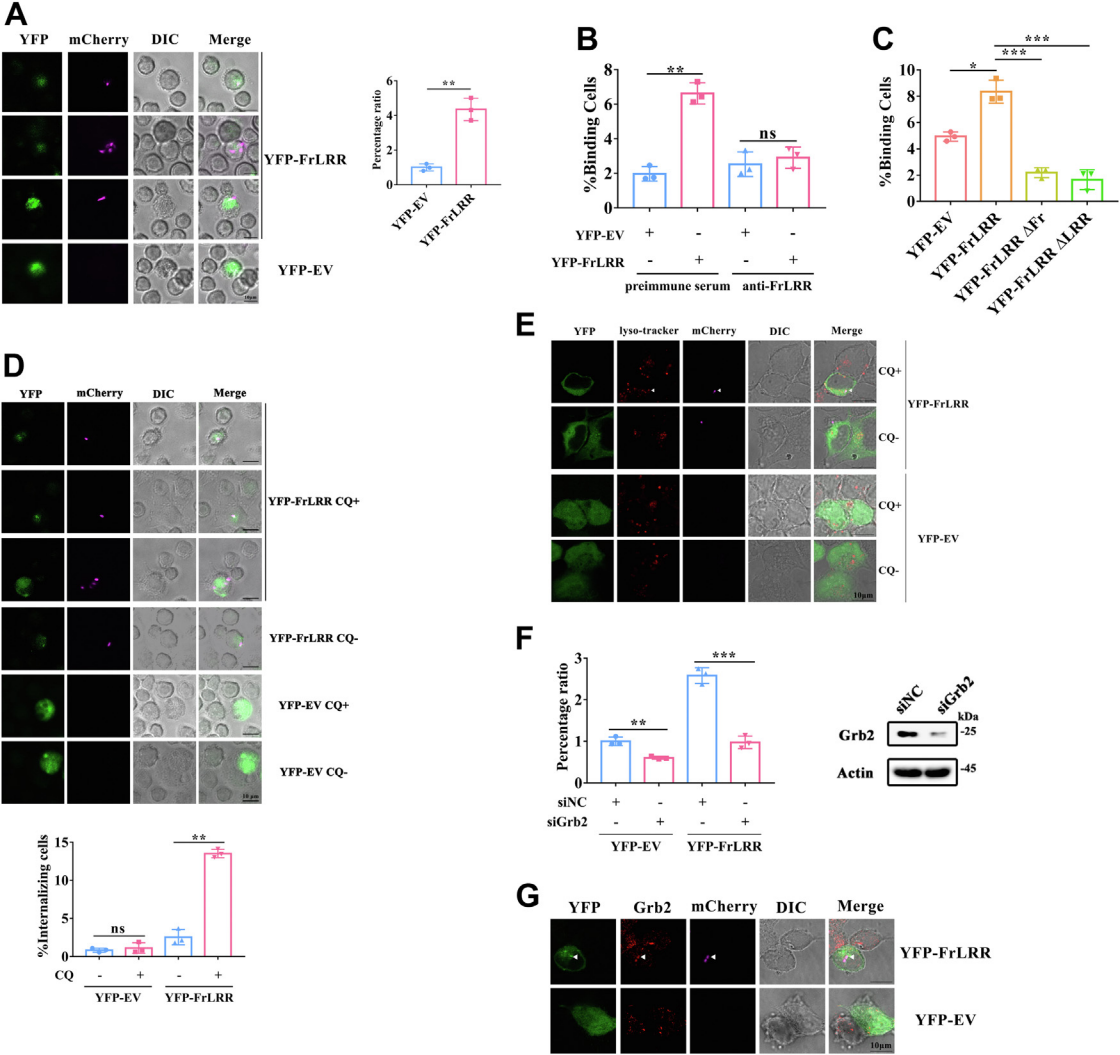
**FrLRR enhances bacterial binding and phagocytosis.***A*, analysis of the *E. coli* binding capability of U-937 cells expressing FrLRR. Cells transfected with empty vector YFP and vector encoding YFP-FrLRR were incubated with *E. coli* expressing mCherry for 1 h, and the fluorescence was observed and imaged under a fluorescence microscope. Scale bars, 10 μm. The statistical graph shows the ratio of the percentage of *E. coli* binding cells for YFP-FrLRR over the percentage of *E. coli* binding cells for YFP-EV. *p* = 0.006. Data are representative of three biological replicates, and the numbers of cells analysed for each experiment (FrLRR *versus* EV) were 400 *versus* 385, 466 *versus* 430, and 445 *versus* 392. *B*, analysis of the blocking effect of anti-FrLRR antibody on *E. coli* binding capability of U-937 cells expressing FrLRR. Cells transfected with vector encoding YFP-FrLRR were incubated with anti-FrLRR antibody or preimmune serum at 37 °C for 1 h and then incubated with *E. coli* expressing mCherry for 1 h. The fluorescence was observed and imaged under a fluorescence microscope. The statistical graph shows the frequency of U-937 cells binding *E. coli*. *P* = 0.0051 (preimmune serum) and 0.3478 (anti-FrLRR antibody). Data are representative of three biological replicates, and the numbers of cells analyzed for each experiment (preimmune-FrLRR vs preimmune-EV vs antibody-FrLRR *versus* antibody-EV) were 400 *versus* 326 *versus* 315 *versus* 270, 556 *versus* 315 *versus* 325 *versus* 269, and 535 *versus* 398 *versus* 322 *versus* 349. *C*, the roles of Fr domain and LRR region in the bacterial binding of FrLRR. Cells transfected with empty vector YFP, vector encoding YFP-FrLRR, YFP-FrLRR ΔFr, or YFP-FrLRR ΔLRR were incubated with *E. coli* expressing mCherry for 1 h. The fluorescence was observed and imaged under a fluorescence microscope. The statistics graph showed the frequency of U-937 cells binding *E. coli*. *p* = 0.0391 (YFP-EV *versus* YFP-FrLRR), 0.0004 (YFP-EV *versus* YFP-FrLRR ΔFr) and 0.0006 (YFP-EV *versus* YFP-FrLRR ΔLRR). Data are representative of three biological replicates, and the numbers of cells analyzed for each experiment (EV *versus* FrLRR *versus* ΔFr *versus* ΔLRR) were 157 *versus* 171 *versus* 251 *versus* 286, 160 *versus* 140 *versus* 284 *versus* 254 and 184 *versus* 179 *versus* 247 *versus* 285. *D*, confocal imaging of bacterial binding of U-937 cells expressing FrLRR after the inhibition of lysosome activity by CQ. Cells transfected with vector encoding YFP-FrLRR were divided into two groups: untreated (CQ−) and treated with 50 μM CQ (CQ+) at 37 °C for 3 h and then incubated with *E. coli* expressing mCherry for 1 h. Fluorescence was observed and imaged under a fluorescence microscope. Scale bars, 10 μm. The statistical graph is based on the fluorescence results. *p* = 0.0015 (YFP-FrLRR) and 0.5519 (YFP-EV). Data are representative of three biological replicates, and the numbers of cells analyzed for each experiment (FrLRR-CQ+ *versus* FrLRR-CQ- *versus* EV-CQ+ *versus* EV-CQ-) were 262 *versus* 234 *versus* 208 *versus* 230, 265 *versus* 272 *versus* 161 *versus* 182, and 264 *versus* 332 *versus* 239 *versus* 174. *E*, confocal imaging of FrLRR (*green*), lysosome (*red*) and *E. coli* (mCherry, *magenta*) colocalization in representative HEK293T cells treated with 50 μM CQ (CQ+) in 37 °C for 3 h or untreated (CQ-). Scale bars, 10 μm. *F*, U-937 cells were transfected with negative control siRNA (siNC) or Grb2 siRNA (siGrb2) and YFP-FrLRR and then incubated with *E. coli* expressing mCherry as mentioned above. The statistical graph shows the *E. coli*-binding cell percentage ratio (the ratio for YFP-EV with siNC was set as 1). *p* = 0.0003 (YFP-FrLRR) and 0.0034 (YFP-EV). Data are representative of three biological replicates, and the numbers of cells analyzed for each experiment (FrLRR-siGrb2 *versus* FrLRR-siNC *versus* EV-siGrb2 *versus* EV-siNC) were 237 *versus* 306 *versus* 262 *versus* 122, 215 *versus* 221 *versus* 275 *versus* 142, and 214 *versus* 201 *versus* 283 *versus* 182. *G*, Confocal imaging of FrLRR (*green*), Grb2 (*red*), and *E. coli* (mCherry, *magenta*) colocalization in representative HEK293T cells. *White arrowheads* indicate colocalization of FrLRR, Grb2 and *E. coli*. Scale bars, 10 μm ns, no significance; ∗*p* < 0.05, ∗∗*p* < 0.01, ∗∗∗*p* < 0.001 (two tailed, unpaired Student’s *t* test). The data are presented as the mean (± SD). Data are representatives of at least two independent experiments. CQ, chloroquine; FrLRR, Fr domain-containing leucine-rich repeat receptor; FrLRR ΔFr, truncated FrLRR deleting the Fr domain; FrLRR ΔLRR, truncated FrLRR deleting the LRR region that starts from LRRNT to LRRCT.

Many proteins containing LRR motifs can bind to a wide range of pathogens and are the primary receptors for innate immune responses in animals and plants. To explore the potential immune function of FrLRR, we conducted a bacterial binding experiment. U-937 cells transfected with YFP-FrLRR plasmids were incubated with *E. coli* DE3 (BL21) expressing mCherry proteins that emit red fluorescence. Microscopic observation and statistical results showed that the bacterial binding percentage of YFP-FrLRR-expressing cells was more than three times that of negative control cells (Fig. 5*A*). Jurkat TAg cells transfected with FLAG-FrLRR (FLAG-tag-FrLRR) also displayed a significantly higher bacterial binding (52.71%) than those transfected with empty vector (36.89%) (Fig. S14).

To verify the role of FrLRR in *E. coli* binding, we used preimmune serum or anti-FrLRR antibody to block membrane-localized FrLRR expressed by U-937 cells in bacterial binding experiments. The statistical results showed that when treated with preimmune serum, the *E. coli* binding of cells expressing YFP-FrLRR was significantly higher than that of cells expressing empty vector; however, after treatment with anti-FrLRR antibody, there was no significant difference in the binding percentage between these two groups (Fig. 5*B*). These results indicate that FrLRR enhances bacterial binding and may directly bind to the pathogens.

To investigate the contributions of the Fr domain and the LRR region to the function of FrLRR, we constructed two deletion mutants, FrLRR ΔFr with Fr domain (from 34aa to 165aa) deletion and FrLRR ΔLRR with LRR region (from 188aa to 397aa) deletion (Fig. S2), and then tested them in U-937 cells by binding experiments. The results showed that the *E. coli* binding of YFP-EV (empty vector), YFP-FrLRR, YFP-FrLRR ΔFr, and YFP-FrLRR ΔLRR was 4.72%, 8.35%, 2.19%, and 1.66%, respectively (Fig. 5*C*), indicating that both the Fr domain and the LRR region play vital roles in full-length FrLRR-mediated bacterial binding.

Combining the above result with that in Figure 4*H*, which showed cytoplasmic FrLRR had a lysosome localization, we hypothesized that FrLRR might mediate cell phagocytosis of bacteria. To test this hypothesis, we examined the cytoplasmic localization of FrLRR and *E. coli* in the presence of chloroquine (CQ), which inhibits intracellular lysosome activity. We found that with CQ treatment, the percentage of U-937 cells with mCherry-labeled *E. coli* cytoplasmic localization, which indicates phagocytic cells, was increased in FrLRR-transfected cells (Fig. 5*D*). The percentage of phagocytic cells with CQ treatment (∼13.15%) was significantly higher than that of the control group (∼2.54%). Upon CQ treatment, the colocalization FrLRR with *E. coli* in the lysosomes of HEK293T cells was observed (Figs. 5*E* and S15). These results indicate that membrane FrLRR may bind and could mediate the delivery of extracellular bacteria into lysosomes through receptor-mediated phagocytosis, suggesting FrLRR plays a role in pathogen defense.

Because FrLRR was found in the BbGrb2 complex and Grb2 is involved in cellular phagocytosis (11, 51), we examined whether Grb2 is involved in FrLRR-mediated bacterial phagocytosis in U-937 cells. We found that even when Grb2 was only slightly knocked down by siRNA, it remarkably reduced the percentage of phagocytotic cells by ∼3-fold in FrLRR-transfected cells (Fig. 5*F*). Interestingly, siGrb2 also reduced the phagocytotic percentage of control cells by ∼1.7-fold. The colocalization of FrLRR with Grb2 and *E. coli* was also observed in HEK293T cells (Fig. 5*G*). Thus, FrLRR promotes bacterial phagocytosis through Grb2.

### Slit2-N mediates a direct bacterial binding

**Figure 6:**
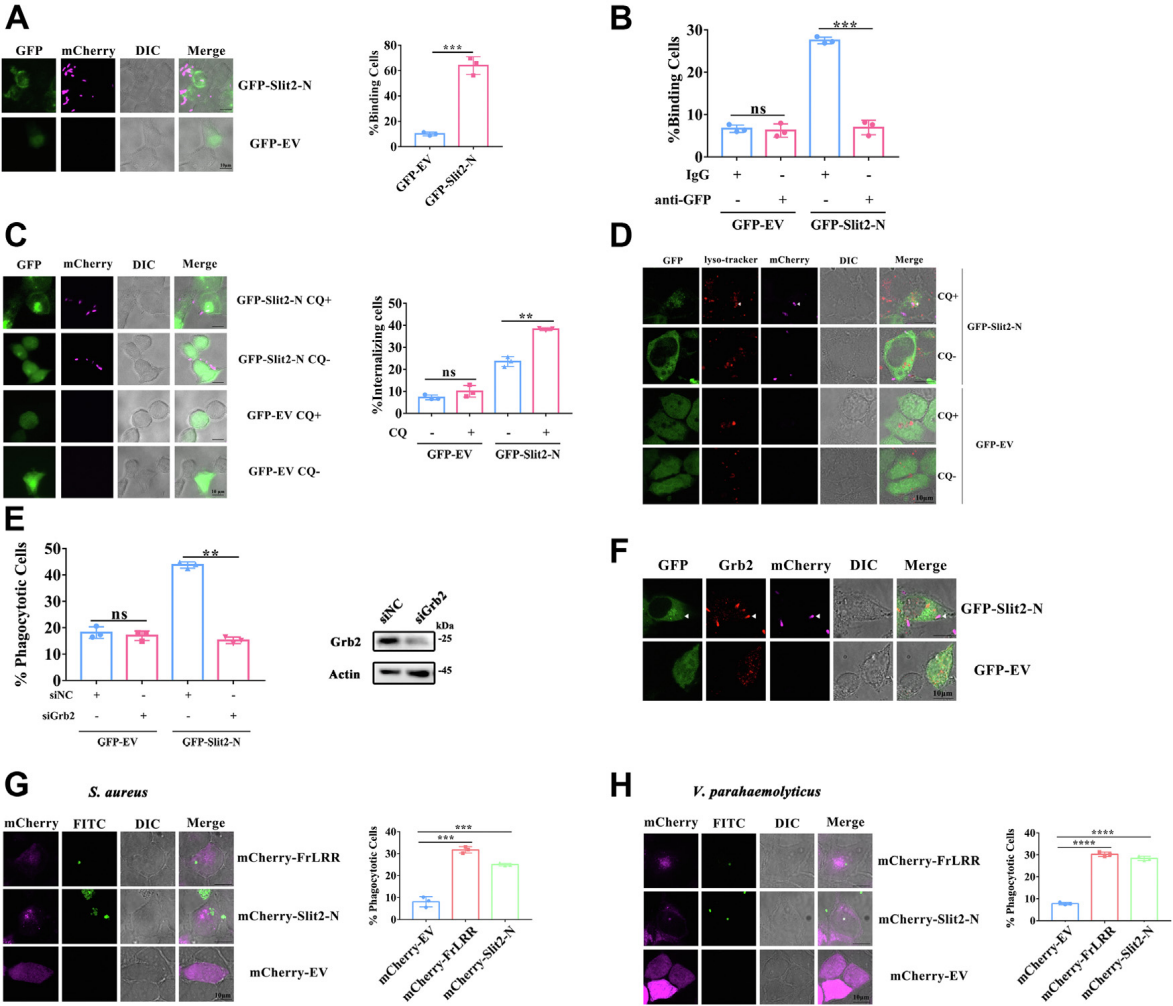
**Slit2-N mediates a direct bacterial binding.***A*, HEK293T cells expressing Slit2-N gained the capability to bind *E. coli*. Cells transfected with empty vector encoding GFP or vector encoding GFP-Slit2-N were incubated with *E. coli* expressing mCherry for 1 h, and the fluorescence was observed and imaged under a fluorescence microscope. Scale bars, 100 μm. The statistical graph shows the frequency of HEK293T cells binding to *E. coli*. *p* = 0.0002. Data are representative of three biological replicates, and the numbers of cells analyzed for each experiment (Slit2-N *versus* EV) were 123 *versus* 79, 105 *versus* 81, and 129 *versus* 69. *B*, the anti-GFP antibody blocked GFP-Slit2-N, which attenuated the ability of cells to bind *E. coli*. Cells transfected with vector encoding GFP-Slit2-N were incubated with anti-GFP antibody or IgG at 37 °C for 1 h and then incubated with *E. coli* expressing mCherry for 1 h. The fluorescence was observed and imaged under a fluorescence microscope. The statistical graph shows the frequency of HEK293T cells binding *E. coli*. *p* = 0.0004 (GFP-Slit2-N) and 0.6996 (GFP-EV). Data are representative of three biological replicates, and the numbers of cells analyzed for each experiment (Sli2-N-preimmune *versus* Sli2-N-antibody *versus* EV-preimmune *versus* EV-antibody) were 305 *versus* 327 *versus* 315 *versus* 263, 229 *versus* 260 *versus* 317 *versus* 346, and 254 *versus* 183 *versus* 222 *versus* 263. *C*, the number of Slit2-N-transfected HEK293T cells internalizing *E. coli* increased after lysosome activity was inhibited by CQ. The cells transfected with vector encoding GFP-Slit2-N were divided into two groups, which were untreated (CQ-) and treated with 50 μM CQ (CQ+) at 37 °C for 3 h and then incubated with *E. coli* expressing mCherry for 1 h. The fluorescence was observed and imaged under a fluorescence microscope. Scale bars: 10 μm. The statistical graph is based on the fluorescence results. *p* = 0.0079 (GFP-Slit2-N) and 0.1662 (GFP-EV). Data are representative of three biological replicates, and the numbers of cells analyzed for each experiment (Slit2-N-CQ+ *versus* Slit2-N-CQ- *versus* EV-CQ+ *versus* EV-CQ-) were 120 *versus* 121 *versus* 117 *versus* 209, 106 *versus* 164 *versus* 116 *versus* 184, and 117 *versus* 128 *versus* 116 *versus* 153. *D*, confocal imaging of Slit2-N (*green*), lysosome (*red*) and *E. coli* (mCherry, *magenta*) colocalization in representative HEK293T cells treated with 50 μM CQ (CQ+) in 37 °C for 3 h or untreated (CQ-). Scale bars, 10 μm. *E*, HEK293T cells were transfected with negative control siRNA (siNC) or Grb2 siRNA (siGrb2) and GFP-Slit2-N and then incubated with *E. coli* expressing mCherry as mentioned above. The statistical graph shows the frequency of HEK293T cells binding to *E. coli*. *p* = 0.0086 (GFP-Slit2-N) and 0.5047 (GFP-EV). Data are representative of three biological replicates, and the numbers of cells analyzed for each experiment (Slit2-N-siGrb2 *versus* Slit2-N-siNC *versus* EV-siGrb2 *versus* EV-siNC) were 91 *versus* 86 *versus* 94 *versus* 97, 79 *versus* 104 *versus* 94 *versus* 92, and 100 *versus* 95 *versus* 117 *versus* 97. *F*, confocal imaging of Slit2-N (*green*), Grb2 (*red*), and *E. coli* (mCherry, *magenta*) colocalization in representative HEK293T cells. Scale bars, 10 μm. *G*, analysis the capability to bind *S. aureus* of human HEK293T cells expressing FrLRR or Slit2-N. Cells transfected with empty vector encoding mCherry or vector encoding mCherry-FrLRR or mCherry-Slit2-N were incubated with FITC (fluorescein isothiocyanate isomer)-labeled *S. aureus* for 2 h, and the fluorescence was observed and imaged under a fluorescence microscope. Scale bars, 10 μm. The statistical graph shows the frequency of HEK293T cells binding to *S. aureus*. *p* = 0.0001 and 0.0003 (mCherry-EV *versus* mCherry-FrLRR or mCherry-Slit2-N). Data are representative of three biological replicates, and the numbers of cells analyzed for each experiment (mCherry-EV *versus* mCherry-FrLRR *versus* mCherry-Slit2-N) were 216 *versus* 241 *versus* 133, 282 *versus* 232 *versus* 171, and 206 *versus* 220 *versus* 175. *H*, analysis of the capability to bind *V. parahaemolyticus* of HEK293T cells expressing FrLRR or human Slit2-N. Cells transfected with empty vector encoding mCherry or vector encoding mCherry-FrLRR or mCherry-Slit2-N were incubated with FITC-labeled *V. parahaemolyticus* for 2 h, and the fluorescence was observed and imaged under a fluorescence microscope. Scale bars, 10 μm. The statistical graph shows the frequency of HEK293T cells binding to *V. parahaemolyticus*. *p* < 0.0001 (mCherry-EV *versus* mCherry-FrLRR or mCherry-Slit2-N). Data are representative of three biological replicates, and the numbers of cells analyzed for each experiment (mCherry-EV *versus* mCherry-FrLRR *versus* mCherry-Slit2-N) were 262 *versus* 253 *versus* 265, 274 *versus* 262 *versus* 310, and 274 *versus* 298 *versus* 297. ns, no significance; ∗∗*p* < 0.01, ∗∗∗*p* < 0.001, ∗∗∗∗*p* < 0.0001 (two-tailed, unpaired Student’s *t* test). The data are presented as the mean (± SD). Data are representatives of at least two independent experiments. FrLRR, Fr domain-containing leucine-rich repeat receptor; GFP-Slit2-N, GFP-tagged Slit2-N; siGrb2, Grb2 siRNA; siNC, negative control siRNA.

The protein with the highest sequence identity to the LRR region of FrLRR in *B. belcheri* is BbSlit2; thus, we explored whether its homologous protein human Slit2-N exhibits the function of the bacterial binding. The experimental results showed that HEK293T cells expressing GFP-Slit2-N displayed a significantly higher percentage (65.39%) of cells binding *E. coli* than those expressing GFP-EV (9.72%) (Fig. 6*A*), indicating that Slit2-N mediates bacterial binding. To verify this, we used IgG or anti-GFP antibody to block GFP-Slit2-N expressed by HEK293T cells and then analyzed the effect on bacterial binding. The statistical results showed that in cells expressing GFP-Slit2-N, the binding percentage of cells treated with anti-GFP antibody was significantly lower than that of cells treated with IgG antibody, whereas in cells transfected with empty vector, there was no significant difference in the binding percentage between the two antibody treatments (Fig. 6*B*). This result indicated that Slit2-N may directly bind to the bacteria to mediate cellular bacterial binding.

Next, we examined whether Slit2-N mediates phagocytosis in HEK293T cells. We found that when lysosomal activity was inhibited by CQ, mCherry intensity was increased on both the surface and inside of the cells transfected with Slit2-N (Fig. 6*C*). In addition, we observed that Slit2-N colocalized with some *E. coli* in the lysosomes (Figs. 6*D* and S15). These results indicate that Slit2-N could mediate the delivery of extracellular bacteria into lysosomes. We next tested whether Grb2 is also involved in the bacterial phagocytosis function of Slit2-N in HEK293T cells. The results showed that Grb2 silencing remarkably reduced the percentage of bacteria-phagocytotic cells expressing GFP-Slit2-N (Fig. 6*E*). We also observed the colocalization of Slit2-N with Grb2 and *E. coli* (Fig. 6*F*). These results confirm the contribution of Grb2 to bacterial phagocytosis mediated by Slit2-N. The above result in Figure 5*C* shows that the LRR region was not sufficient but was essential for mediating bacterial binding. The predicted 3D structure revealed that the four LRR regions of Slit2-N also form a curved solenoid shape (Fig. S16). Together, these results suggest that Slit2-N plays a role in defending against bacterial infection and that Slit2-type LRR region-containing proteins might represent a novel type of immune receptor.

In addition to *E. coli*, we checked if FrLRR and Slit2-N could enhance the binding of other species of bacteria. We found that both of them efficiently promoted *Staphylococcus aureus* (*S. aureus*) (Figs. 6*G* and S17) and *Vibrio parahaemolyticus* (*V. parahaemolyticus*) (Figs. 6*H* and S17) binding to HEK293T cells. Thus, FrLRR and Slit2-N promote multiple species of bacteria binding to cells.

## Discussion

In this study, using amphioxus *B. belcheri* BbGrb2 as bait, we identified a novel *B. belcheri* immune receptor, FrLRR, which is primarily expressed in intestinal cells. Consisting of an Fr domain, a Slit2-type LRR region, and a GPI anchor site at the end of the C-terminus, FrLRR exhibits a predicted curved solenoid structure resembling VLRs. Functionally, FrLRR may directly bind to bacteria and promote phagocytosis *via* Grb2. Additionally, for the first time, we also demonstrate the possible role of human Slit2-N in bacterial elimination through direct binding to bacteria. Therefore, our study suggests that Slit2-type LRR region-containing LRR proteins might represent a novel type of immune receptor.

The adaptor molecule Grb2 plays an essential role in orchestrating actin remodeling during phagocytosis, which is an actin-dependent process mediated by receptor clustering upon detection of the particle to be ingested, and receptor tyrosine phosphorylation (7, 11, 52, 53). Our findings that FrLRR coimmunoprecipitates with Grb2 and promotes bacterial binding and phagocytosis through Grb2 suggest FrLRR may be a novel type of phagocytic receptor. However, we currently still do not know how FrLRR interacts with Grb2 without a cytoplasmic tail. A possible explanation is that they interact indirectly and an unknown mediator such as a coreceptor might mediate their association. Just like in TCR signaling, TCRα/β chains do not have cytoplasmic signaling tails, but they can form a complex with the CD3 coreceptors which harbor signaling tails to transduce signaling by recruiting downstream adaptors and signaling proteins including Grb2 (4, 12). Another example is the FcγRIIIb, one type of three classified groups of FcγR, is a GPI-linked receptor lacking a cytoplasmic tail (54). However, FcγRIIIb is capable of signaling to initiate phagocytosis by cooperation with other phagocytic receptors, such as FcγRIIA and integrins (55). Moreover, we could not exclude the possible involvement of other phagocytic receptors signaling which might be activated by FrLRR expression. Indeed, our mass spectrum analysis detected some peptides from several receptors, such as interleukin-18 receptor 1-like and platelet endothelial aggregation receptor 1-like, in BbGrb2 immunoprecipitates. To find if one of these receptors may work as coreceptor with FrLRR will help us to understand the mechanisms of FrLRR-mediated phagocytosis and the evolution of immune signaling pathway.

Recently, Slit2 has been reported to enhance the ability of M1-TAMs (tumor-associated macrophages) to phagocytose tumor cells, which occurred *via* suppressing the production of IL6, a cytokine can increase macrophage fibrosis and reduce phagocytosis, from breast cancer cells or macrophages in the tumor microenvironment (31). The literature has revealed a molecular mechanism that regulates the phenotypic plasticity of TAMs of Slit2 (31). Here, we also showed that Slit2N promotes phagocytosis, but different from the indirect regulation of M1-TAMs phagocytosis by Slit2, we have discovered that Slit2-N may bind directly to bacteria and then activate the target particles/phagocytotic receptor axis. The different findings between our study and the literature (31) is because we have analyzed phagocytosis in different environments and conditions. Just like FrLRR, Slit2-N could not directly bind with Grb2; meanwhile, the HEK293 cell line, which has undetectable expression of Robos or Dscam which are canonical receptors for Slit2-N (56, 57), was used to test Slit2-N-mediated phagocytosis, therefore Slit2-N might employ a novel coreceptor to activate the phagocytotic receptor/Grb2 axis. To analyze the receptors revealed in Grb2 immunoprecipitates may supply critical clues. Although the detailed mechanisms are unclear, our study has established the potential role of Slit2-N as an innate immune protein in the elimination of bacteria.

Although FrLRR and BbFR are approximately 700 k nucleotides apart on the same scaffold and evidence of gene duplication may have been lost, the possibility of an association between them still exists, considering that the distance between hFR4 and the other three hFRs on chromosome 11 is approximately 22 MB and they have evolved from a common ancestral gene (58). The existence of this novel and unique *B. belcheri* gene FrLRR represents an example of exon shuffling being an effective method to form genes encoding proteins with new functions in evolution (59).

VLR is a lymphocyte receptor found in jawless vertebrates that generate diversity through gene conversion, but its origin remains unknown (18). The crystal structure of a VLR-like protein Bf66946 primarily expressed in the gill of *B. floridae* has been resolved and resembles the lamprey VLRC, implying the emergence of VLR-like molecules in basal chordates (60). Here, we show that FrLRR is predominantly expressed in intestinal cells, has motifs and protein structures similar to those of VLRs in agnatic vertebrates, and functions as an immune receptor. The gill or intestine expression pattern of these two VLR-like genes implies that diverse adaptive immune receptors might be produced in these tissues and cells, similar to VLRs (61). However, neither of these two genes (Bf66946 and FrLRR) seems to be generated by somatic recombination, and whether chordate animals have adaptive immunity remains an enigma.

In conclusion, our study revealed a novel amphioxus *B. belcheri* immune receptor, FrLRR, and demonstrated a previously undescribed potential role for Slit2-N in bacterial biding and elimination, implying that a Slit2-type LRR region containing protein may play an innate immune receptor. Further exploration of the ligand of FrLRR and the detailed mechanisms of Slit2-N in phagocytosis will deepen our understanding of these new immune receptors.

## Experimental procedures

### Animals, plasmids, and antibodies

*B. belcheri* was collected from Zhanjiang of Guangdong Province in China and raised in seawater. BALB/c mice were bred in the Laboratory Animal Center of Sun Yat-sen University. The animal experiments were performed according to guidelines approved by the Animal Care and Ethics Committee of Sun Yat-Sen University.

Nucleic acid and protein sequences were obtained from the NCBI (https://www.ncbi.nlm.nih.gov/protein/) and LanceletDB (http://genome.bucm.edu.cn/lancelet/index.php) databases. Human Grb2 (GenBank, NP_002077.1) was amplified from Jurkat TAg cell cDNA and cloned into the pcDNA3.1-FLAG vector (Invitrogen). The BbGrb2 and FrLRR sequences were cloned from Chinese amphioxus (*B. belcheri*) gut cDNA. The scrambled control siRNA and the siGrb2 were synthesized by Ruibiotech Co, Ltd. The primers and siRNA used in this article are shown in Table S1. The predicted 3D structures of proteins were predicted by the Phyre2 software.

Antibodies against HA (Y-11), GFP/YFP (B-2), and actin (I-19) were purchased from Santa Cruz Biotechnology. Antibodies specific for Na/K-ATPase (3010) and GAPDH (glyceraldehyde-3-phosphate dehydrogenase) (2118) were from Cell Signaling Technology. Horseradish peroxidase (HRP)-conjugated secondary antibodies were from Jackson ImmunoResearch. The M2 antibody against FLAG (F3165) was purchased from Sigma–Aldrich. Alexa Fluor 488–coupled chicken anti-mouse (A-21200), Alexa Fluor 594–coupled chicken anti-rabbit (A-21442), and Alexa Fluor 647–coupled donkey anti-mouse (A-31571) secondary antibodies were purchased from Invitrogen.

### Extraction of total RNA, synthesis of cDNA, PCR cloning, and Quantitative real-time PCR

The instructions of the MagZol Reagent kit from Magen were used to extract total RNA from various tissues of amphioxus *B. belcheri*.

Reverse transcription PCR was used to synthesize cDNA according to the guidelines of the Promega GoScript Reverse Transcription System. In brief, experimental RNA was combined with the experimental primers, and then the mix was thermally denatured at 70 °C for 5 min and chilled on ice. A reverse transcription reaction mix was assembled on ice containing nuclease-free water, reaction buffer, reverse transcriptase, magnesium chloride, dNTPs (deoxy-ribonucleoside triphosphate) (PCR Nucleotide Mix), and ribonuclease inhibitor. As a final step, the template-primer combination was added to the reaction mix on ice. Following an initial annealing at 25 °C for 5 min, the reaction was incubated at 42 °C for up to 1 h. Because no cleanup or dilution is necessary following cDNA synthesis, the product was directly added to the amplification reactions.

FrLRRwas cloned from amphioxus *B. belcheri* gut cDNA using KOD-Plus-Neo DNA polymerase (TOYOBO). The primers used are shown in Table S1. The target DNA band above the 1000 bp DNA ladder (Thermo Fisher) was separated by agarose gel electrophoresis and purified using the Magen HiPure Gel DNA Kit. The purified PCR products were ligated to the pGEM-T vector using T4 DNA Ligase (Promega). The ligation products were transformed into *E.coli*DH5α, which was then cultured overnight to obtain bacterial colonies with positive PCR results confirmed by PCR and agarose gel electrophoresis. The plasmids were isolated from these colonies cultured overnight using a Magen HiPure Plasmid Kit and then sent for sequencing.

Quantitative real-time PCR was performed in a total volume of 10 μl with 5 μl of 2× RealStar Green Power Mixture (Genstar), 0.2 μl of primers, 0.5 μl of cDNA templates and double-distilled water. Real-time PCR was performed in a LightCycler 480 System (Roche). Data were collected and quantified using the 2^−ΔΔCt^ method according to the Ct values of target genes and normalized to endogenous 18S RNA as a control.

### Preparation of polyclonal antibodies

Recombinant target proteins were prepared as the antigens. Briefly, cDNA encoding BbGrb2 or FrLRR was inserted into pET-28a+. The recombinant plasmid was transfected into DE3 (an *E. coli* strain) cells for protein expression. The recombinant his-tagged protein was purified by binding to a Ni Sepharose-preloaded column and eluted using elution buffer (pH 7.4) containing 20 mM Na_3_PO_4_, 0.5 M NaCl, and 250 mM imidazole. The recombinant protein concentration was measured using the BCA and Bradford assays (Bio–Rad).

The BALB/c mice immunization procedure was performed as previously described (3). Briefly, four mice were first intraperitoneally injected with the antigen protein mixed with an equal volume of Freund’s complete adjuvant (Sigma). After 14 days, the mice were injected a second time with the antigen protein mixed with incomplete Freund’s adjuvant (IFA, Sigma) and then immunized using the same method every week until the antibody titer met the threshold for subsequent experiments. The animal study was reviewed and approved by the Animal Care and Ethics Committee of Sun Yat-Sen University.

### Mass spectrometry

Protein samples were prepared for mass spectrometry measurements as previously described (62). Briefly, the indicated protein complex was subjected to SDS–PAGE. When the electrophoresis was carried out, the sample became a compact slit-like band after loading, the protein band was excised from a Coomassie-stained SDS–PAGE gel and fragmented into small pieces. Thereafter, the proteins were reduced using 10 mM dithiothreitol for 30 min at 55 °C, followed by alkylation with 55 mM iodoacetamide for 30 min at room temperature in the dark. Overnight digestion at 37 °C was performed with 0.6 μg of trypsin (Proteomics-grade, Promega) in 25 mM NH_4_HCO_3_. The resulting peptides were acidified using 0.1% formic acid (FA) to stop trypsin digestion (63). After the desalting procedure, the peptides were analyzed by the LTQ Orbitrap Elite system and Easy-nLC 1000 system (Thermo Fisher Scientific).

### Cell culture and transfection

Jurkat T cells and U-937 cells were cultured in RPMI-1640 complete medium (HyClone) supplemented with 10% and 5% fetal bovine serum (FBS; HyClone), respectively. HEK293T cells were cultured in high-glucose Dulbecco’s modified Eagle’s minimal medium complete medium (HyClone) supplemented with 10% FBS. Before transfection, the cells were resuspended in Opti-MEM (Gibco). Jurkat T cells were electrotransfected with plasmids using nucleofection (Lonza 4D Nucleofector system). HEK293T cells were transfected using Lipofectamine3000 (Invitrogen) or polyethyleneimine. Cell lines were authenticated using short tandem repeat profiling at GENEWIZ, Inc . The *mycoplasma* test for cell culture was performed on a monthly basis using a PCR *Mycoplasma* Detection Kit (abm, G238). Cells used in experiments were within ten passages from thawing.

### IP assay and Western blotting

Cells were harvested and lysed in lysis buffer containing 20 mM Tris-HCl (pH 7.5), 150 mM NaCl, 5 mM EDTA (pH 8.0), 5 mM sodium diphosphate, 1 mM sodium orthovanadate (Na_3_VO_4_), 1 mM PMSF, 1% Nonidet P-40, 10 μg/ml aprotinin, and 10 μg/ml leupeptin. After centrifugation at 13,200*g* at 4 °C to remove cellular debris, the supernatant was incubated with the indicated antibody overnight at 4 °C, and the proteins were immunoprecipitated using protein G-Sepharose (GE Healthcare) for an additional 4 h at 4 °C. The precipitated proteins or total cell lysates were separated by SDS–PAGE, and proteins were transferred to polyvinylidene fluoride (PVDF) membranes at 400 mA. The membranes were blocked in 4% bovine serum albumin (BSA) (dissolved in Tris buffered saline (1 M, pH 7.5)-with 1% Tween 20 buffer) and then incubated with the indicated primary antibody overnight at 4 °C, followed by incubation with HRP-conjugated secondary antibodies for 1 h at room temperature. Immunoblots were developed using X-ray film or a ChemiDoc instrument (Bio–Rad).

### Preparation and immunohistochemical staining of tissue sections

Adults of the Chinese amphioxus *B. belcheri* were collected and kept in filtered seawater for 2 days. The animals were then sacrificed and cut at 1-cm intervals. The obtained tissue blocks were fixed in 4% paraformaldehyde in PBS, dehydrated in graded ethanol, embedded in paraffin, and systematically cut into 8-μm histological sections the following day. Tissue blocks were cut transversally and mounted onto glass slides. The sections were dewaxed, rehydrated, absterged, bleached with 3% H_2_O_2_ to inhibit endogenous enzyme activity, and heated in 10 mM citric acid recovery buffer (pH 6.0) for antigen retrieval. The sections were then blocked in 5% skim milk in PBS for 1 h and incubated with primary antibodies at 4 °C overnight. The sections were then incubated with horseradish peroxidase-conjugated secondary antibodies for 1 h at room temperature, followed by colorimetric detection using DAB (BOSTER), and counterstaining with hematoxylin. After dehydration and transparency, the sections were mounted in a neutral resinous mounting medium. Images were captured using a ZEISS Lumar.V12 system.

### Cell immunofluorescence staining and organelle staining

Jurkat T cells or amphioxus *B. belcheri* gut cells were fixed in 4% paraformaldehyde (PFA, Sigma–Aldrich), layered onto Poly-L-Lysine (PLL, Sigma–Aldrich)-coated microscope slides, and permeabilized with 0.2% Triton X-100. After blocking with 2% BSA in PBS for 30 min, the cells were stained overnight at 4 °C with the indicated primary antibodies. The cells were washed with PBS three times, after which fluorescent dye-conjugated secondary antibodies were added and incubated for 1 h at room temperature for double staining. Nuclei were counterstained with 4′,6-diamidino-2-phenylindole (1 μg/ml). The organelle-specific fluorescent dyes LysoTracker-L7526, MitoTracker-M22426, ER-Tracker-E12353 (Thermo Fisher Scientific) were used to stain lysosomes, ER, and mitochondria according to the manufacturer’s guidelines. As a negative control, secondary antibodies were used alone. After three washes with PBS, the cells were mounted with a drop of mounting medium (Mowiol 4-88, Sigma–Aldrich). Images were acquired using a Leica SP5 laser-scanning confocal microscope equipped with a 63× objective lens with laser excitation at 405 nm, 488 nm, 561 nm, or 633 nm.

### Subcellular fractionation

Cells washed in ice-cold PBS were resuspended in ice-cold hypotonic buffer (10 mM HEPES, pH 7.4, 42 mM KCl, 5 mM MgCl_2_, 1 mM DTT, and protease inhibitors [leupeptin, PMSF, and aprotinin]) for 20 min on ice. The cell suspension was passed through a 30-gauge needle ten times, followed by centrifugation at 200*g* for 10 min. The pellet was used as the nuclear fraction. The supernatant was centrifuged at 25,000*g* for 1 h, and the pellet was used as the plasma membrane.

### Bacterial binding assay

U-937 cells (induced by PMA for 48 h) or HEK293T cells were transfected with the indicated plasmids, cultured in 24-well plates on coverslips, and then incubated with mCherry-conjugated *E. coli* in fresh medium at 37 °C for 1 h. After incubation, the cells were immediately placed on ice to stop the binding assay and phagocytosis and washed with cold phosphate-buffered saline three times. The coverslips were examined by microscopy using a 63× objective lens.

## Data availability

All data are contained within the manuscript and supporting information.

## Supporting information

This article contains supporting information.

## Conflict of interest

The authors declare that they have no conflicts of interest with the contents of this article.
