# Supplementary material for: Identification of a novel amphioxus leucine-rich repeat receptor involved in phagocytosis reveals a role for Slit2-N-type LRR in bacterial elimination
Source: J Biol Chem. 2023 Apr 10;299(6):104689. doi: 10.1016/j.jbc.2023.104689 (PMC10199209; doi:10.1016/j.jbc.2023.104689)
Supplement: Supporting Figures S1–S17 [file mmc1.docx]

**Identification of a novel amphioxus leucine-rich repeat receptor involved in phagocytosis reveals a role for Slit2-N-type LRR in** **bacterial elimination**

**Yanli Zhan, Chen-si Zhao, Xuemei Qu, Zhi hui Xiao, Chong Deng, Yingqiu Li***

MOE Key Laboratory of Gene Function and Regulation, Guangdong Province Key Laboratory of Pharmaceutical Functional Genes, State Key Laboratory of Biocontrol, School of Life Sciences, Sun Yat-sen University, Guangzhou 510275, China.

***Correspondence:**

Yingqiu Li

[lsslyq@mail.sysu.edu.cn](mailto:lsslyq@mail.sysu.edu.cn)

**Keywords: amphioxus, folate receptor domain (Fr domain)-containing leucine-rich repeat receptor (FrLRR), Grb2, Slit2-N, bacterial binding, phagocytosis**

Supplementary Material

## Supplementary Figures


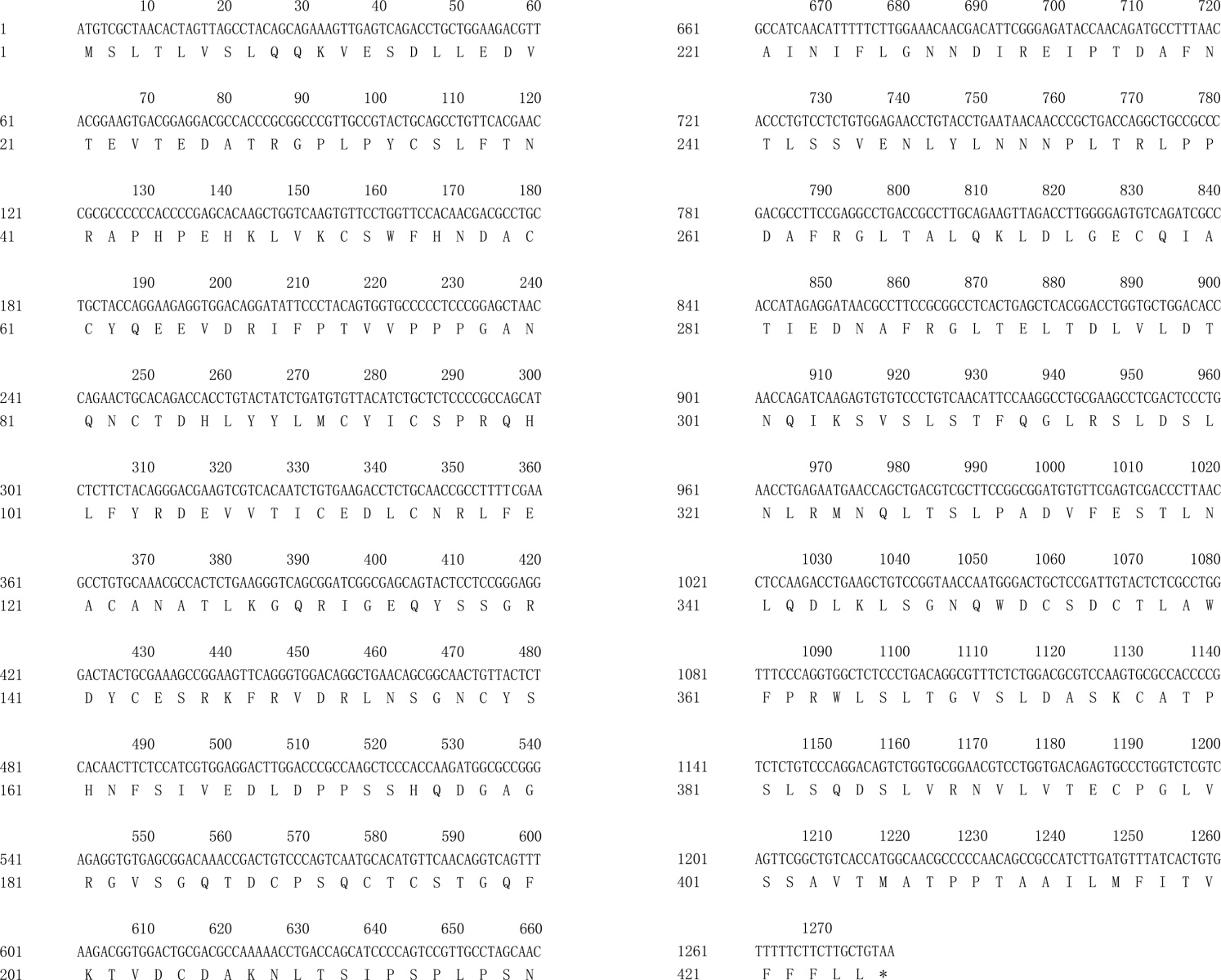


**Supplementary Figure 1.** The nucleic acid sequence and amino acid sequence of FrLRR.


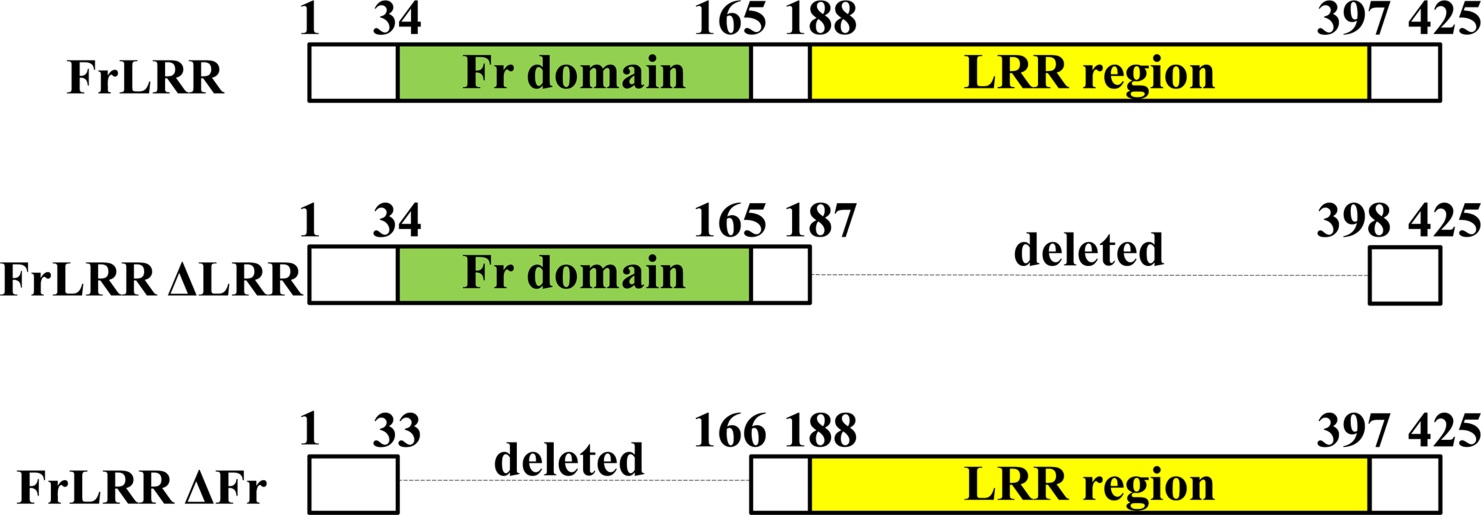


**Supplementary Figure 2.** The schematic diagram of full-length FrLRR, FrLRR ΔFr and FrLRR ΔLRR. The module configuration was predicted by SMART. FrLRR ΔFr, truncated FrLRR deleting the Fr domain; FrLRR ΔLRR, truncated FrLRR deleting the LRR region that starts from LRRNT to LRRCT.


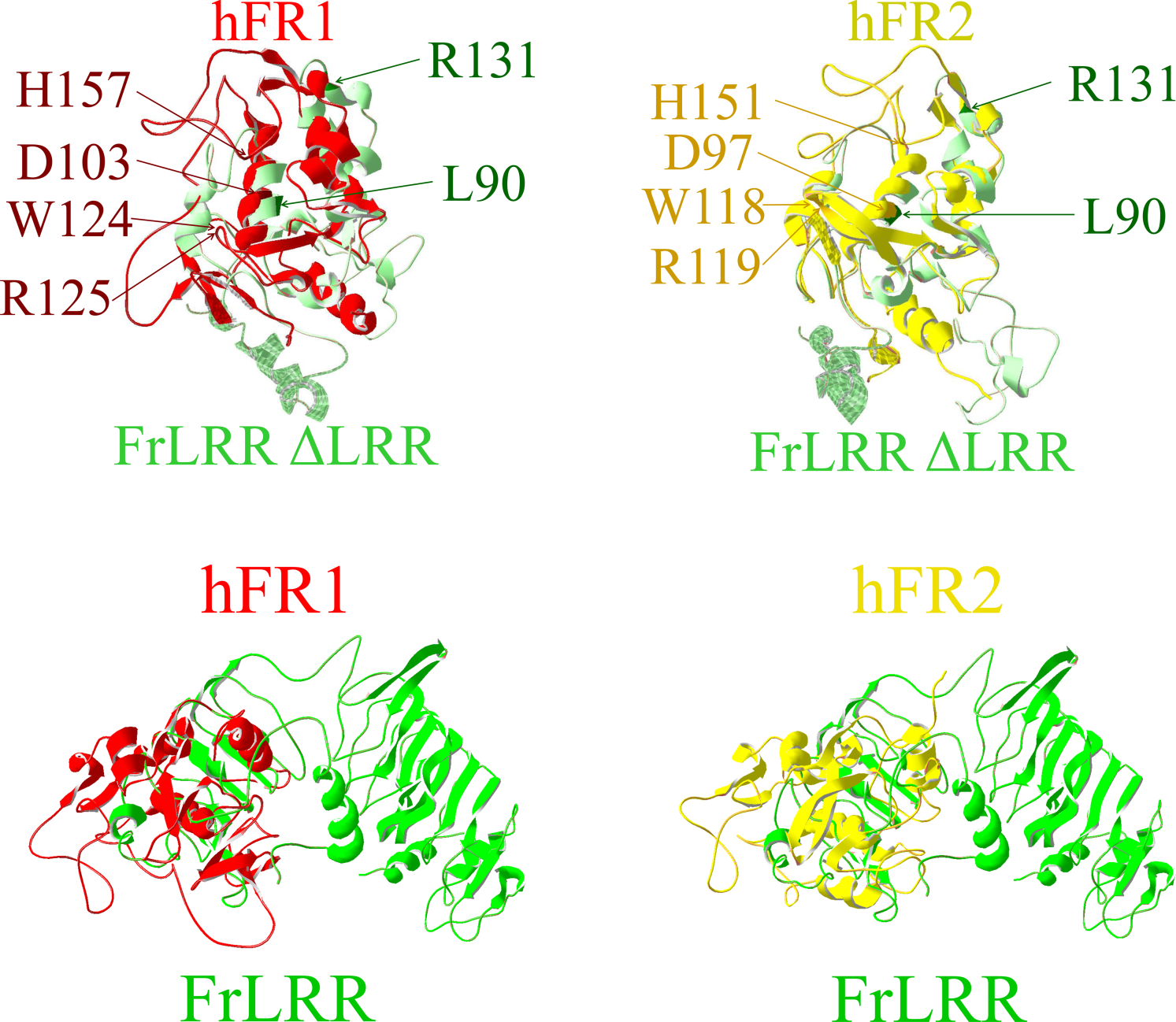


**Supplementary Figure 3.** Superposition of predicted 3D structures of FrLRR ΔLRR (upper panels) or full-length FrLRR (lower panels) and hFR1/2 by the Phyre2 software. The residues labeled in dark red or yellow mean two of the four amino acids in hFR1 or hFR2 that form the key hydrogen bond with folate, and those in dark green indicate the counterpart in FrLRR.


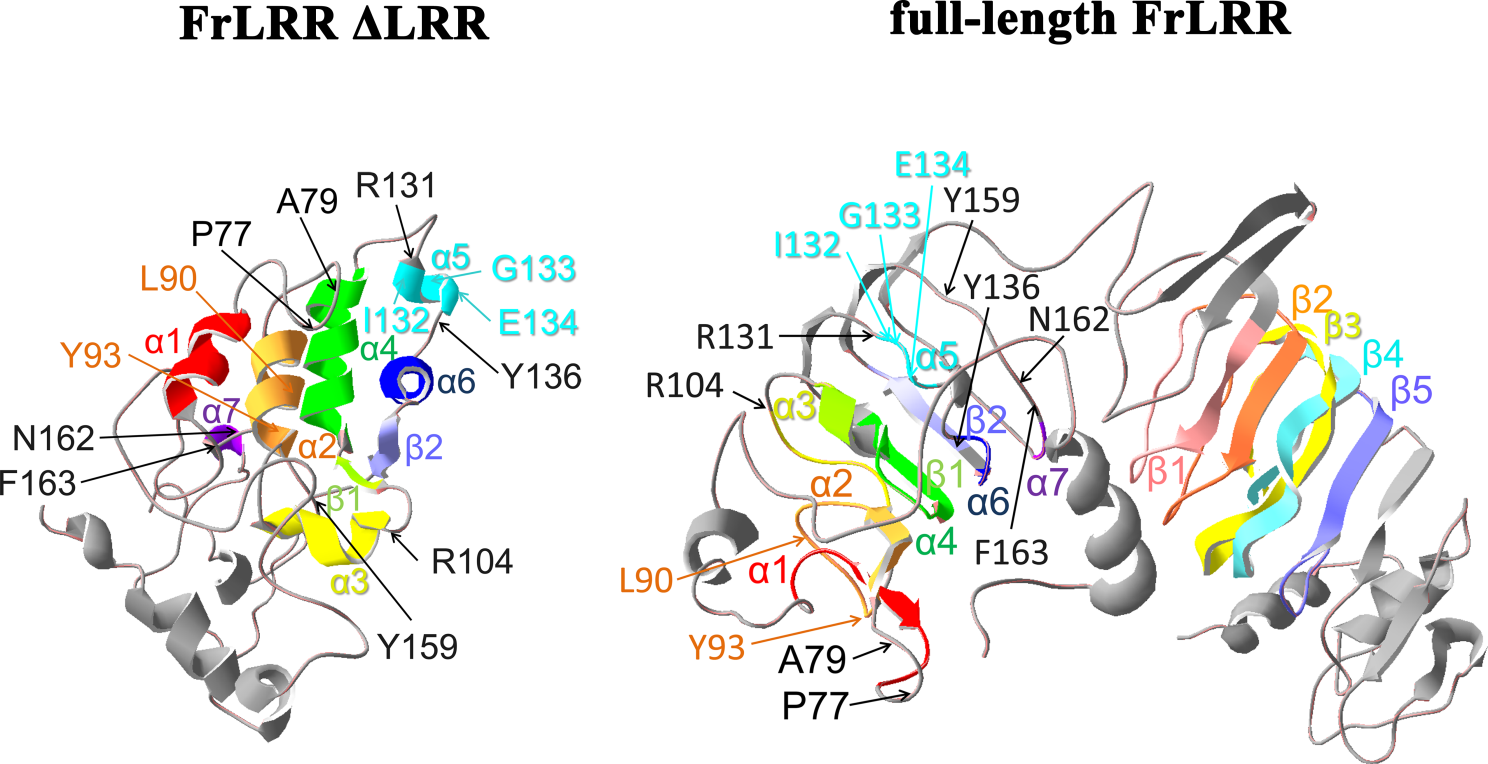


**Supplementary Figure 4.** The positions of essential amino acids for binding folate, corresponding to those in hFR1 and hFR2, in 3D structure of full-length FrLRR and FrLRR ΔLRR. The 3D structure was predicted by the Phyre2 software.


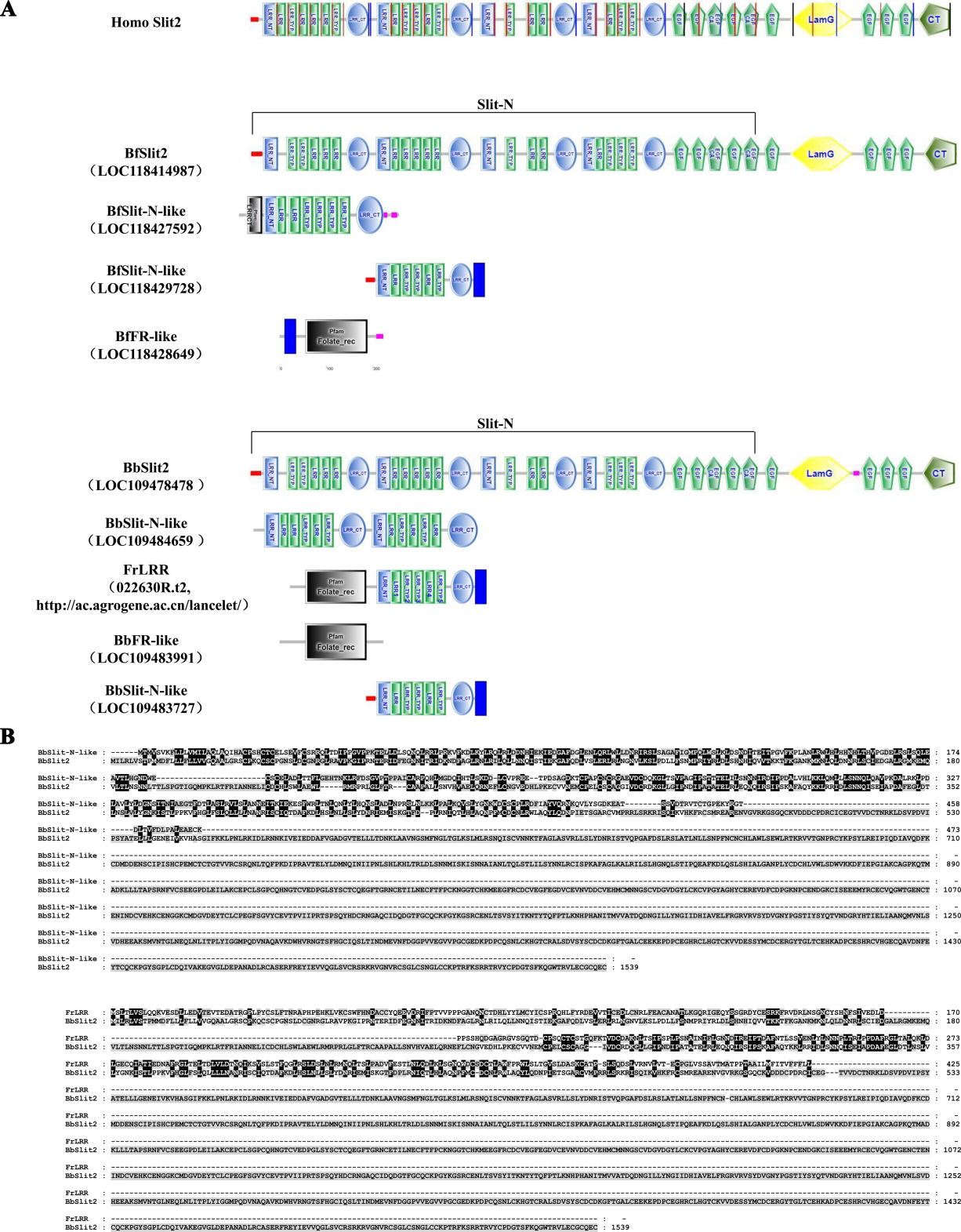


**Supplementary Figure 5.** Domain schematic diagram and amino acid sequence alignment between Slits and FrLRR. (A) Red block, signal peptide; EGF, Epidermal growth factor-like domain; EGF_CA, Calcium-binding EGF-like domain; LamG, Laminin G domain; CT, C-terminal cystine knot-like domain (CTCK); magenta block, Low complexity region; blue block, the hydrophobic segment in the GPI attachment signal sequence or transmembrane region. The vertical lines indicate intron, the colors of the vertical lines indicate the phase of intron (black for 0, blue for 1 and red for 2). (B) BbSlit-N-like (LOC109484659) and FrLRR have the highest sequence identity with BbSlit2 in LRR regions.


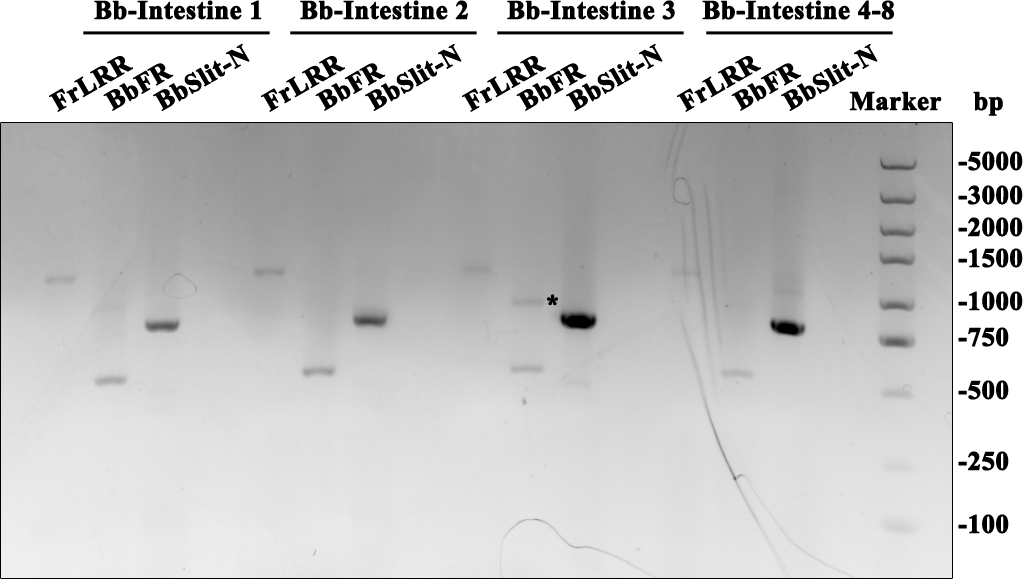


**Supplementary Figure 6.** RT-PCR analysis confirms that all three transcripts BbFR-like, BbSlit2-N-like and FrLRR are expressed in *B. belcheri*. FrLRR is 1278 bp, BbFR-like (LOC109483991) is 666 bp and BbSlit2-N-like (LOC109483727) is 786 bp. The asterisk (*) means non-specific band. Bb-Intestine 1, 2 and 3: RT-PCR was performed with total RNA isolated from intestine of single *B.belcheri*; Bb-Intestine 4-8: RT-PCR was performed with the mixture of total RNA isolated from intestine of five *B.belcheri*. The RT-PCR results of Bb-intestine 2 were shown in figure 3C and reused in this figure to present a complete image and to show that different *B. belcheri* amphioxus individuals have the same pattern of transcripts.


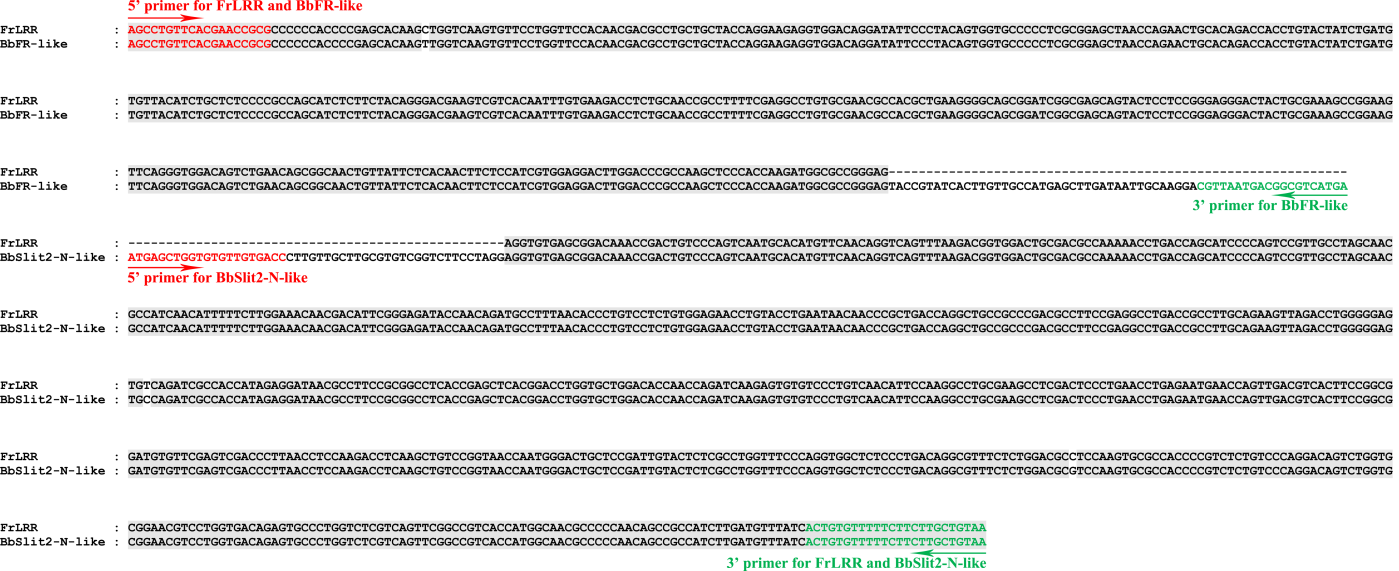


**Supplementary Figure 7.** The sequencing result alignment of RT-PCR analysis in Figure S6. Letters on a gray background indicate identical amino acids; Letters in red (or green) indicate the template sequences for forward (or reverse) primer design.


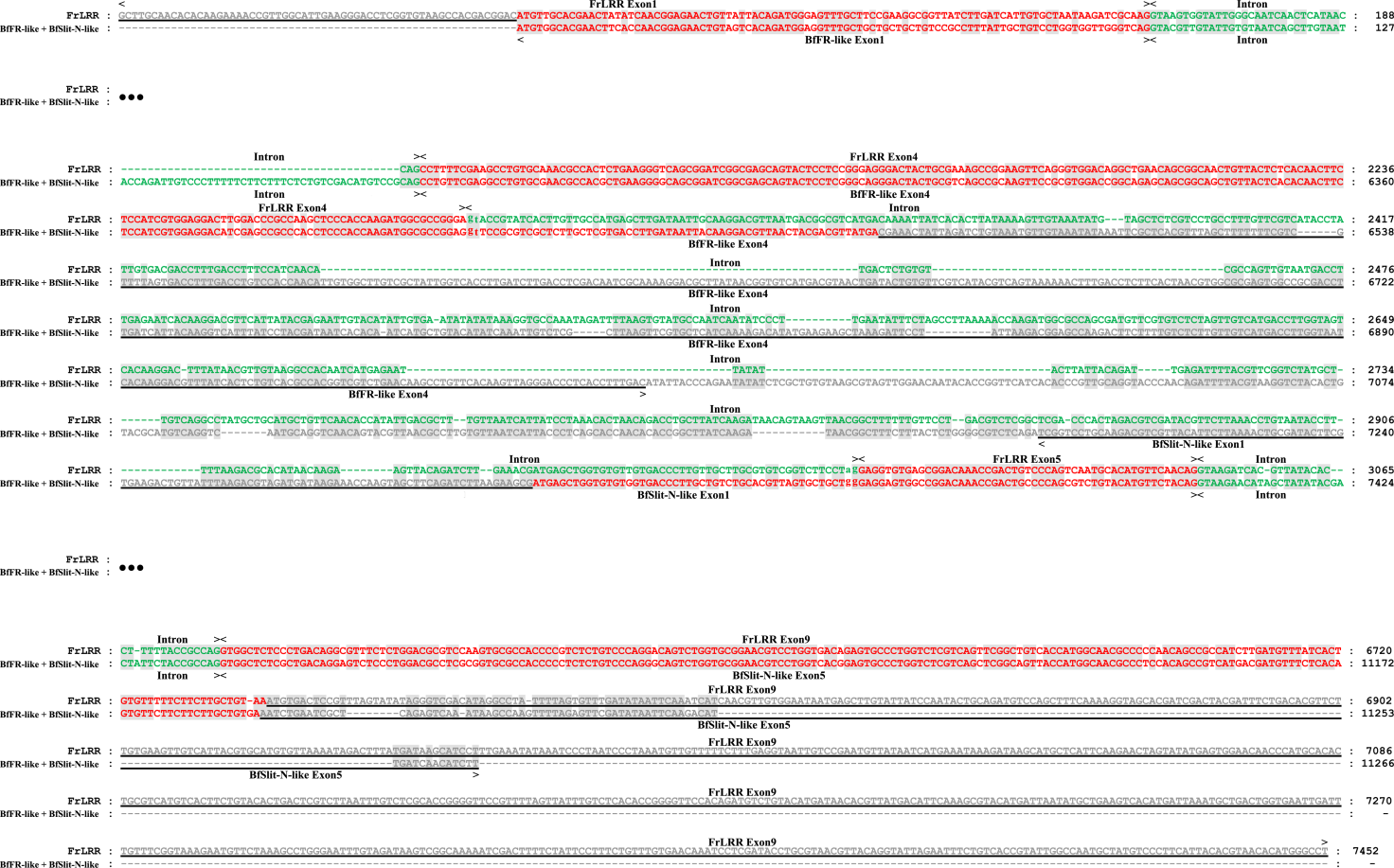


**Supplementary Figure 8.** The partial nucleotide sequence alignment of FrLRR and BfFR-like (LOC118428649) and BfSlit-N-like (LOC118429728). Letters on a gray background indicate identical amino acids; Letters in red indicate the coding sequences; Letters in green indicate the introns; the UTR sequences are underlined in black. The splice donor and acceptor motifs (GT, AG) in the intron of FrLRR between exon 4 and exon 5 and the correspondence nucleic acids in *B. floridae* are indicated by lower case letters.


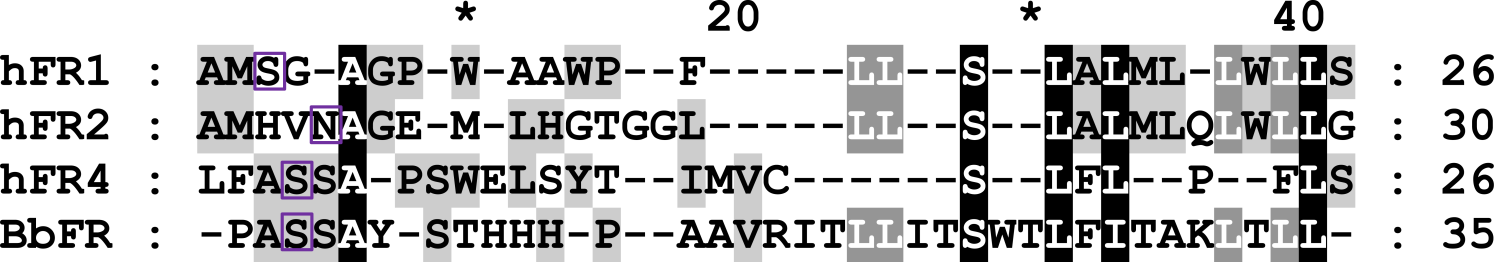


**Supplementary Figure 9.** Comparison of the GPI-anchor site and C-terminal hydrophobic region in BbFR with its counterparts in hFRs. The residues in frame indicate the predicted GPI-anchor cleavage sites with the best score. Letters on a black background indicate identical residues in all four sequences, on a grey background indicate identical residues in two or three sequences.


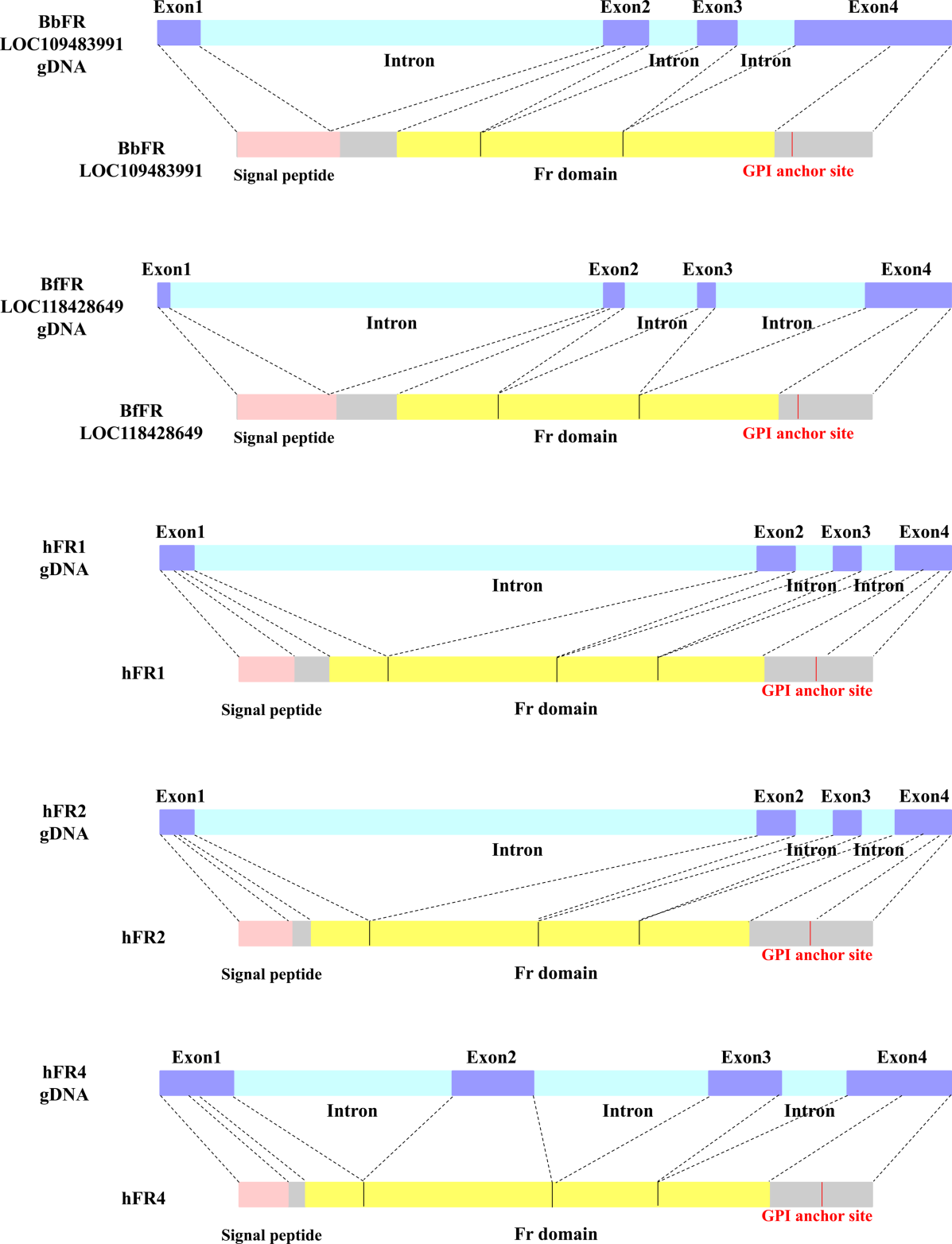


**Supplementary Figure 10.** Schematic diagram of exons encoding amphioxus FRs and human FRs. The slate blue bars mean exons; the light cyan bars mean introns; the light grey bars mean sequences with no functional domains; the yellow bar means the Fr domains; the pink bars mean signal peptides. The GPI anchor sites are marked in red.


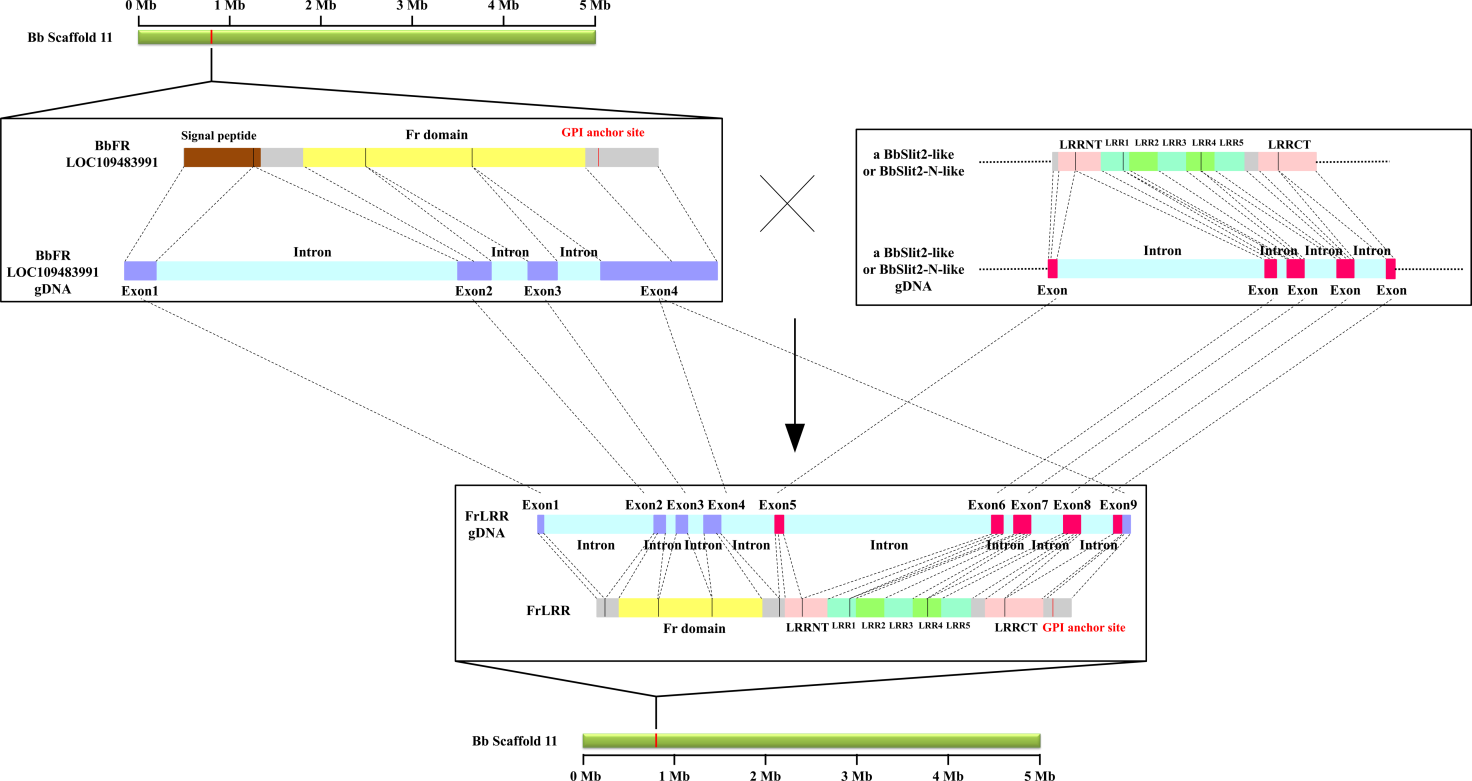


**Supplementary Figure 11.** Schematic depiction of our hypothesis that FrLRR might be generated by exon shuffling of a LRR region of a Slit2-N-like gene into an FR gene in evolution. Several exons (encoding LRR region) from a BbSlit2-like or BbSlit2-N-like gene shuffle into an FR gene locus, which results in the generation of FrLRR gene locus. The green horizontal block represents genomic scaffold 11, in which the red vertical bar indicates the genomic positions of FrLRR. The slate blue and magenta bars mean exons; the light cyan bars mean introns; the light grey bars mean sequences with no predictable functional domains; the yellow bar means the Fr domain; the pink bars mean LRRNT and LRRCT; the light green and cyan bars mean LRRs; the brown bar means signal peptide. The GPI anchor site is marked in red.


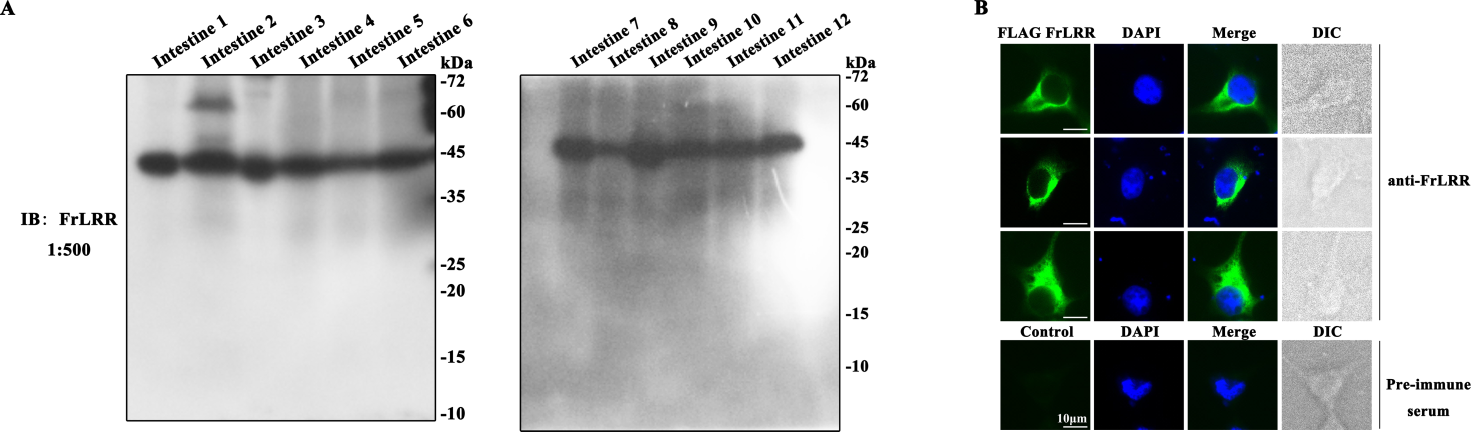


**Supplementary Figure 12.** (A) FrLRR has similar expression levels in the intestines from different *B. belcheri* individuals. The intestinal cells from twelve *B. belcheri* were separately lysed and then immunoblotted with anti-FrLRR antibody. (B) Localization of overexpressed FrLRR in HEK293T cells. After fixation and permeabilization, the cells were incubated with anti-FrLRR antibody, and with pre-immune serum as a control, followed by staining with the secondary antibody as in Figure 4. The images were obtained under a fluorescence microscope.


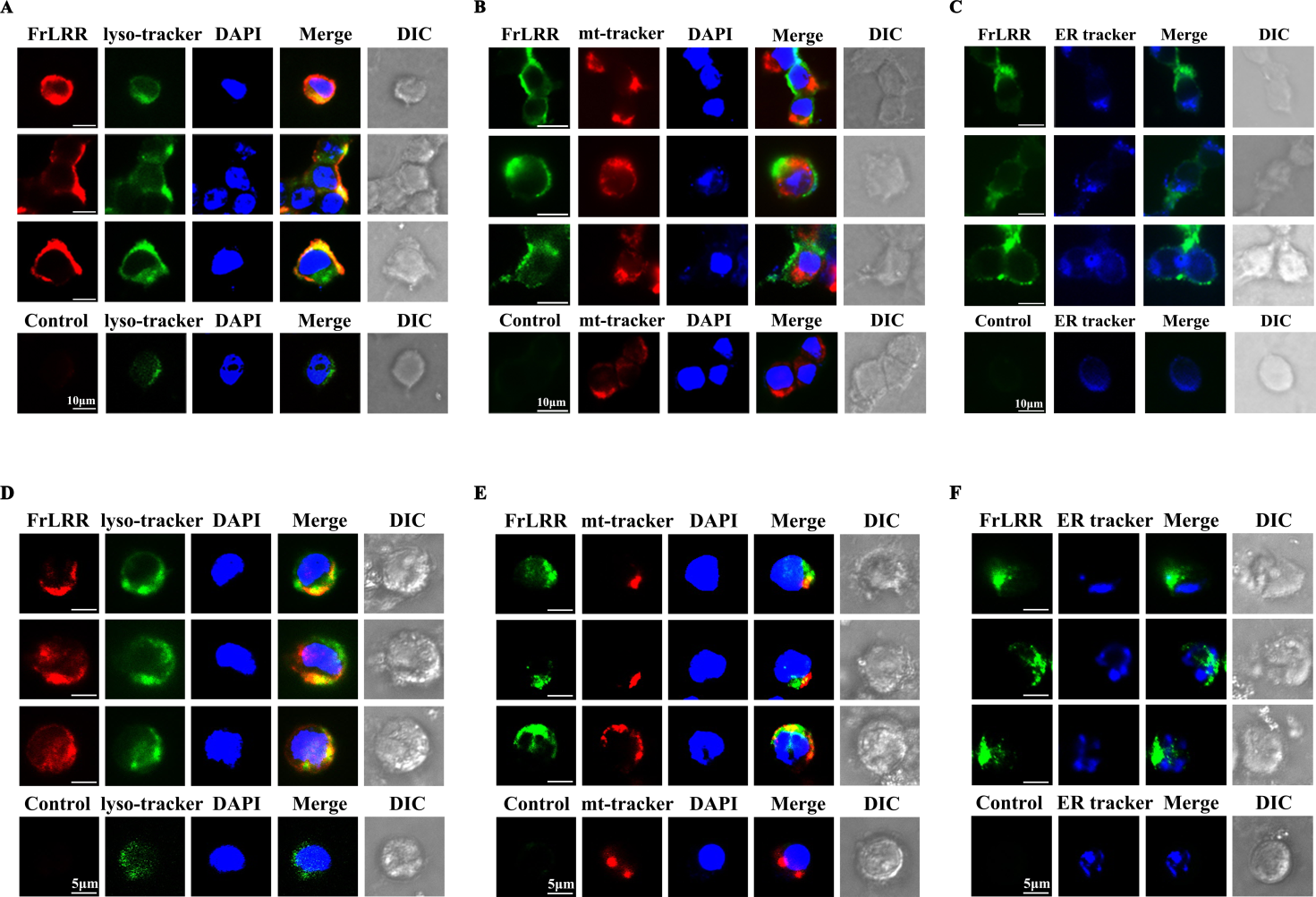


**Supplementary Figure 13.** Immunostaining analysis of the organelle localization for overexpressed FrLRR in HEK293T (A-C) and Jurkat E6.1 (D-F) cells. The cells were transfected with empty vector encoding FLAG or vector encoding FLAG-FrLRR and then separately stained with three organelle dyes according to their guidelines. After fixation and permeabilization, cells were incubated with anti-FrLRR antibody, followed by staining with the secondary antibody as in Figure 4. The images were obtained under a fluorescence microscope.


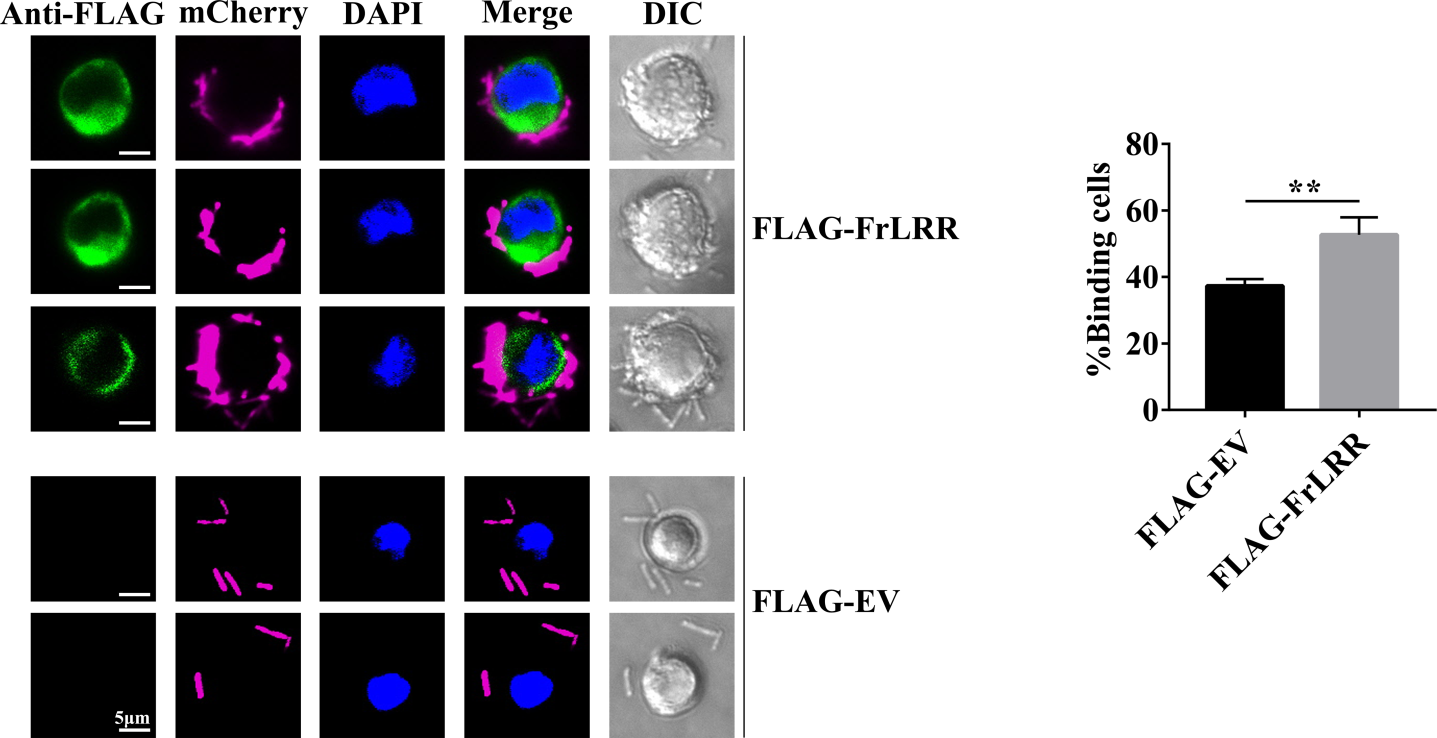


**Supplementary Figure 14.** Analysis of the capability to bind *E. coli* of Jurkat TAg cells expressing FrLRR. Cells transfected with empty vector encoding FLAG or vector encoding FLAG-FrLRR were incubated with *E. coli* expressing mCherry for 1 h, and the fluorescence was observed and imaged under a fluorescence microscope. Scale bars, 5 μm. The statistical graph shows the frequency of Jurkat TAg cells binding to *E. coli*. *p* = 0.0092 ( FLAG-FrLRR versus FLAG-EV, two tailed, unpaired Student’s t-test). Data are representative of three biological replicates (mean ± s. d.), and the numbers of cells analysed for each experiment (FrLRR vs EV) were 133 vs 131, 183 vs 168 and 222 vs 206.


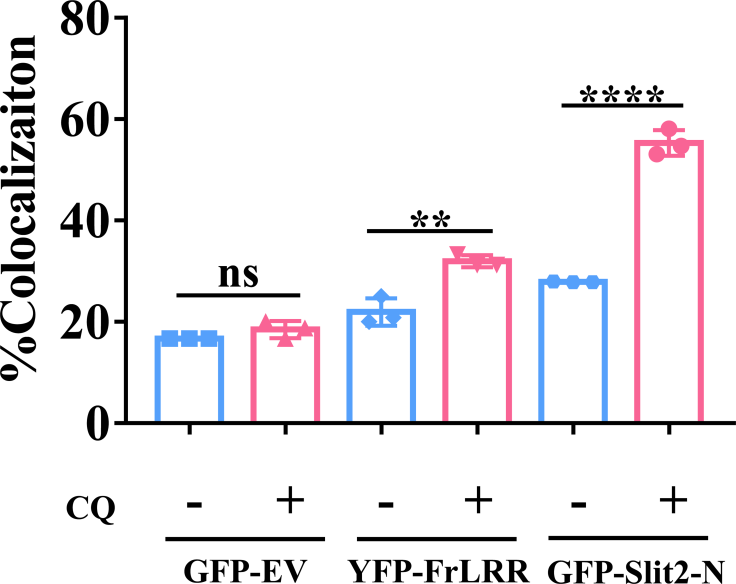


**Supplementary Figure 15.** The statistical graph based on the experiments of Figure 5E and Figure 6D shows the percentages of transfected HEK293T cells with GFP/YFP-tagged proteins colocalizing with *E.coli* (mCherry) in lysosomes. *p* = 0.0041 (YFP-FrLRR), <0.0001 (GFP-Slit2-N) and 0.1369 (GFP-EV). Data are representative of three biological replicates, and the numbers of cells analyzed for each experiment (FrLRR-CQ+ vs FrLRR-CQ- vs Slit2-N-CQ+ vs Slit2-N-CQ- vs EV-CQ+ vs EV-CQ-) were 79 vs 95 vs 77 vs 71 vs 96 vs 70, 78 vs 89 vs 70 vs 75 vs 89 vs 75 and 82 vs 87 vs 79 vs 70 vs 87 vs 73.


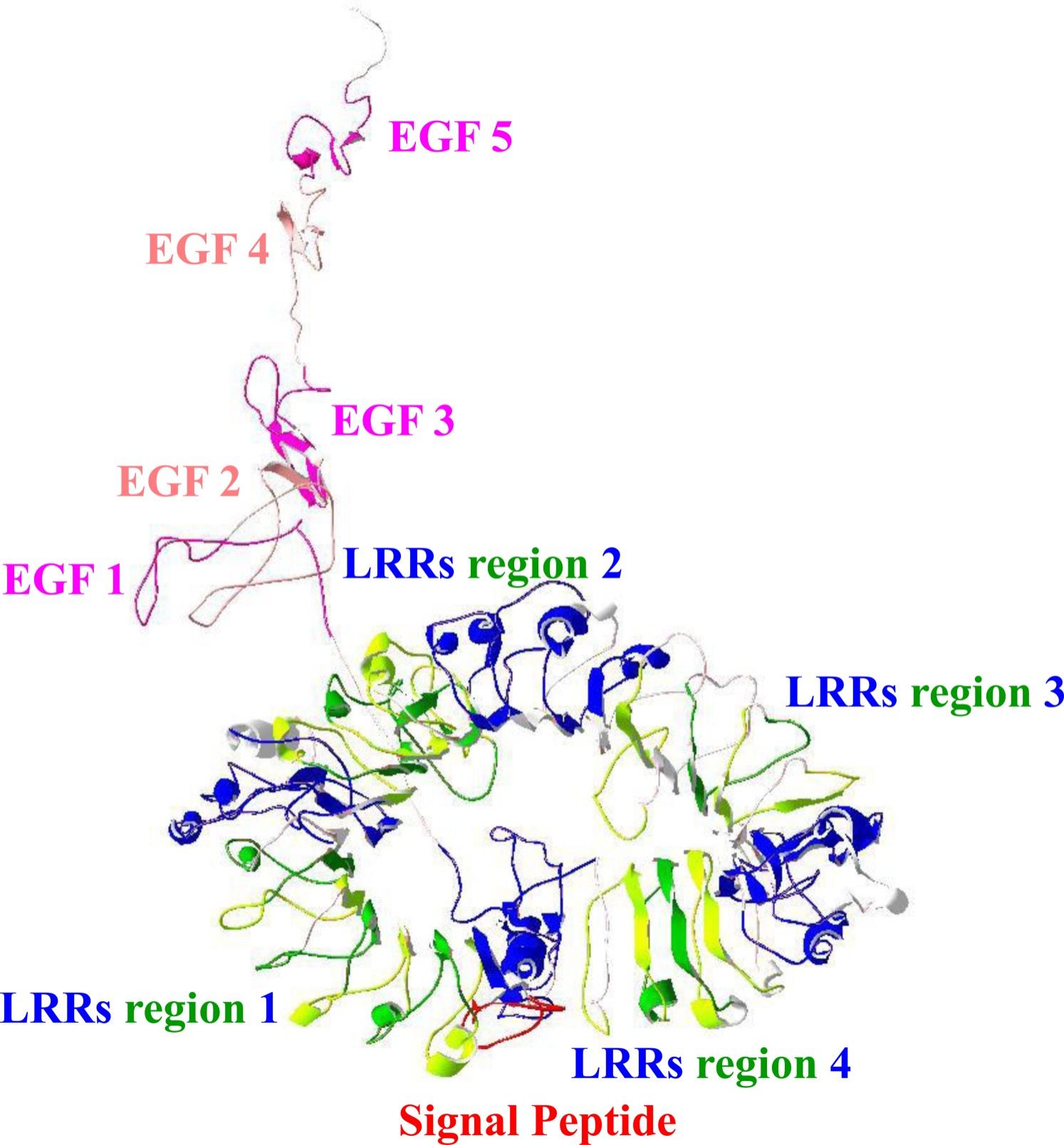


**Supplementary Figure 16.** 3D structure of homo Slit2-N. The 3D structure was predicted by the Phyre2 software.

**
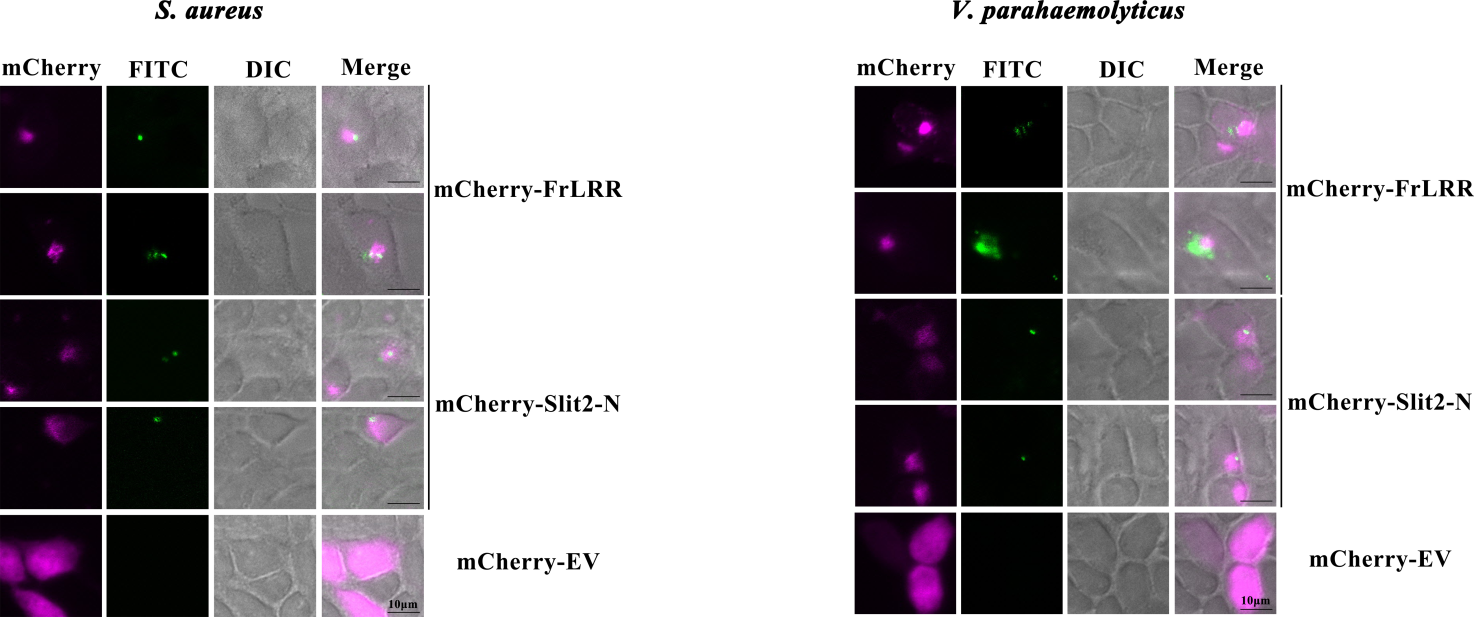
**

**Supplementary Figure 17.** Analysis the capability to bind *S. aureus* and *V. parahaemolyticus* of human HEK293T cells expressing FrLRR or Slit2-N. Cells transfected with empty vector encoding mCherry or vector encoding mCherry-FrLRR or mCherry-Slit2-N were incubated with FITC-labeled *S. aureus* or *V. parahaemolyticus* for 2 h, and the fluorescence was observed and imaged under a microscope. Scale bars, 10 μm.
