# Supplementary material for: Identification of a novel amphioxus leucine-rich repeat receptor involved in phagocytosis reveals a role for Slit2-N-type LRR in bacterial elimination
Source: J Biol Chem. 2023 Apr 10;299(6):104689. doi: 10.1016/j.jbc.2023.104689 (PMC10199209; doi:10.1016/j.jbc.2023.104689)
Supplement: Supporting Table S1 [file mmc2.zip › jbc_00104689_Table S1_mmc2.DOCX]

**Table S1.** Primers and siRNA used in this study.

| Primer and siRNA | Sequences(5’→3’) | Description |
| --- | --- | --- |
| pGEM-T-FrLRR F | ATGAGCCTGTTCACGAACCGCG | Cloning FrLRR into pGEM-T vector |
| pGEM-T-FrLRR R | TTACAGCAAGAAGAAAAACACAGTGA |  |
| pCDNA3.1-FrLRR (HA) F | CCGGAATTCATGAGCCTGTTCACGAACCGCG | Cloning in pCDNA3.1 vector for mammalian expression |
| pCDNA3.1-FrLRR (HA) R | AAGGAAAAAAGCGGCCGCCAGCAAGAAGAAAAACACAGTGA |  |
| pCDNA3.1-FrLRR Delta LRR (HA) S1 | CCGGAATTCATGTCGCTAACACTAGTTAGCCTAC |  |
| pCDNA3.1-FrLRR Delta LRR (HA) A1 | AGCCGAACTGACGAGACCGGTTTGTCCGCTCACACCTCTC |  |
| pCDNA3.1-FrLRR Delta LRR (HA) S2 | GAGAGGTGTGAGCGGACAAACCGGTCTCGTCAGTTCGGCT |  |
| pCDNA3.1-FrLRR Delta LRR (HA) A2 | AAGGAAAAAAGCGGCCGCCAGCAAGAAGAAAAACACAGT |  |
| pFLAG-CMV2-FrLRR (FLAG) F | CCGGAATTCAAGCCTGTTCACGAACCGCG | Cloning into pFLAG CMV2 vector for mammalian expression (FLAG epitope is inserted in front of the N-termination of FrLRR ) |
| pFLAG-CMV2-FrLRR (FLAG) R | CGGGGTACCTTACAGCAAGAAGAAAAACACAGTGA |  |
| pFLAG-CMV2-FrLRR Delta Fr (FLAG) F | AAGGAAAAAAGCGGCCGCGGGGAGAGGTGTGAGCGGAC |  |
| pFLAG-CMV2-FrLRR Delta Fr (FLAG) R | CCGGAATTCTTACAGCAAGAAGAAAAACACAGT |  |
| pEYFP-C1-FrLRR (YFP) F | CCGGAATTCTAGCCTGTTCACGAACCG | Cloning into pEYFP-C1 vector for mammalian expression (EYFP epitope is inserted in front of the N-termination of FrLRR ) |
| pEYFP-C1-FrLRR (YFP) R | CGGGGTACCTTACAGCAAGAAGAAAAACACA |  |
| pEYFP-C1-FrLRR Delta Fr (YFP) F | CCGGAATTCTGGGAGAGGTGTGAGCGGAC |  |
| pEYFP-C1-FrLRR Delta Fr (YFP) R | CGGGGTACCTTACAGCAAGAAGAAAAACACAGT |  |
| Real-time FrLRR F | CCGGGAGAGGTGTGAGCG | For quantitative real time PCR |
| Real-time FrLRR R | ACCGTCTTAAACTGACCTGTTGAA |  |
| Real-time Bbβ-actin F | GGACGTGATCTGACAGACTAC |  |
| Real-time Bbβ-actin R | CCTCTCAGCAGTGGTTGT |  |
| Grb2-specific siRNA | TCAGCCAATTTGTCTCCTA | Interfering and decrease the gene expression of homo sapiens Grb2 |
| BbFR-like AS | TCATGACGCCGTCATTAACG | For BbFR-like (LOC109483991) cloning |
| BbSlit2-N-like S | ATGAGCTGGTGTGTTGTGACC | For BbSlit2-N-like (LOC109483727) cloning |
